# Bony pseudoteeth of extinct pelagic birds (Aves, Odontopterygiformes) formed through a response of bone cells to tooth-specific epithelial signals under unique conditions

**DOI:** 10.1038/s41598-018-31022-3

**Published:** 2018-08-28

**Authors:** Antoine Louchart, Vivian de Buffrénil, Estelle Bourdon, Maïtena Dumont, Laurent Viriot, Jean-Yves Sire

**Affiliations:** 1 CNRS UMR 5242 Institut de Génomique Fonctionnelle de Lyon, Team Evo devo of vertebrate dentition, Université de Lyon, ENS de Lyon, 69364, Lyon cedex, 07 France; 2 Université de Lyon, UCBL, ENSL, CNRS, UMR 5276 LGL-TPE, 69622 Villeurbanne, France; 3 Sorbonne-Universités, Museum National d’Histoire Naturelle, Centre de Recherche sur la Paléobiodiversité et les Paléoenvironnements (CR2P), 75005 Paris, France; 4 The Natural History Museum of Denmark, Section of Biosystematics, DK-2100 Copenhagen, Denmark; 5 Université Pierre et Marie Curie, UMR 7205, Laboratoire Informatique et Systématique, 75005 Paris, France; 6 UMR CNRS/MNHN 7179, “Mécanismes adaptatifs: des organismes aux communautés”, 75005 Paris, France; 7 CNRS UMR7138-Evolution Paris-Seine, Institut de Biologie Paris-Seine, Université Pierre et Marie Curie, 75005 Paris, France

## Abstract

Modern birds (crown group birds, called Neornithes) are toothless; however, the extinct neornithine Odontopterygiformes possessed bone excrescences (pseudoteeth) which resembled teeth, distributed sequentially by size along jaws. The origin of pseudoteeth is enigmatic, but based on recent evidence, including microanatomical and histological analyses, we propose that conserved odontogenetic pathways most probably regulated the development of pseudodentition. The delayed pseudoteeth growth and epithelium keratinization allowed for the existence of a temporal window during which competent osteoblasts could respond to oral epithelial signaling, in place of the no longer present odontoblasts; thus, bony pseudoteeth developed instead of true teeth. Dynamic morphogenetic fields can explain the particular, sequential size distribution of pseudoteeth along the jaws of these birds. Hence, this appears as a new kind of deep homology, by which ancient odontogenetic developmental processes would have controlled the evolution of pseudodentition, structurally different from a true dentition, but morphologically and functionally similar.

## Introduction

**Figure 1:**
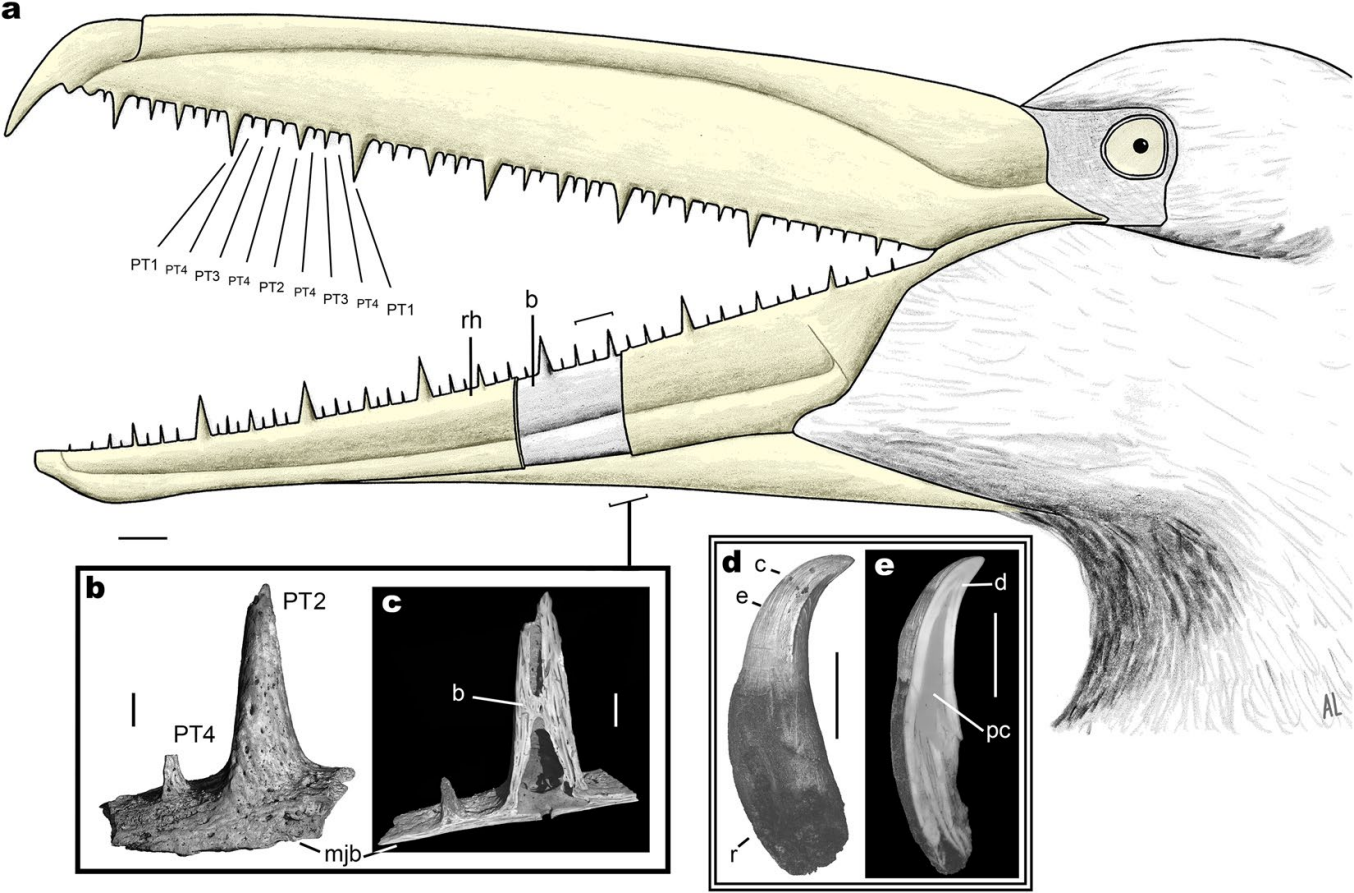
Schematic reconstruction of the pseudodentition of a four-ranked pseudotoothed bird. (**a**) Left view of reconstructed beak and head. The rostrum and most of the mandible are represented with the rhamphotheca (keratinized epithelium; in yellow) covering the jaw bones, except for a small area of the mandible to show the underlying bone. (**b**) Magnification of the bony cores of two adjacent pseudoteeth (the larger is a PT2, the smaller is a PT4) of *Pelagornis mauretanicus* (AaO-PT-B)^5^, in lateral x-ray microtomographic view. (**c**) Structure of the pseudoteeth bony cores in (**a)**, shown in parasagittally truncated x-ray microtomographic view. (**d** and **e**) (double frame), true tooth of the Cretaceous bird *Hesperornis regalis* (YPM.1206B)^22^ for comparison with pseudoteeth (in volume and parasagittally truncated synchrotron x-ray microtomographic views, respectively). Proportions based on data from *Pelagornis* species such as *P. mauretanicus*^5^. Views in inserts are reversed, except (**c**), in order to fit a left mandibular placement in lateral view. The real position (left vs. right, rostral vs. mandibular) of the *P. mauretanicus* pseudoteeth and the *H. regalis* tooth, shown in inserts (**b**–**e**), is indeterminate^5,22^. Distribution of PT1s to PT4s is indicated along part of the rostrum. b, bone; c, crown; d, dentin; e, enamel; mjb, main jaw bone; pc, pulp cavity; r, root; rh, rhamphotheca. Scale bars, main frame 2 cm, inserts (**b**–**e**) 2 mm.

**Figure 2:**
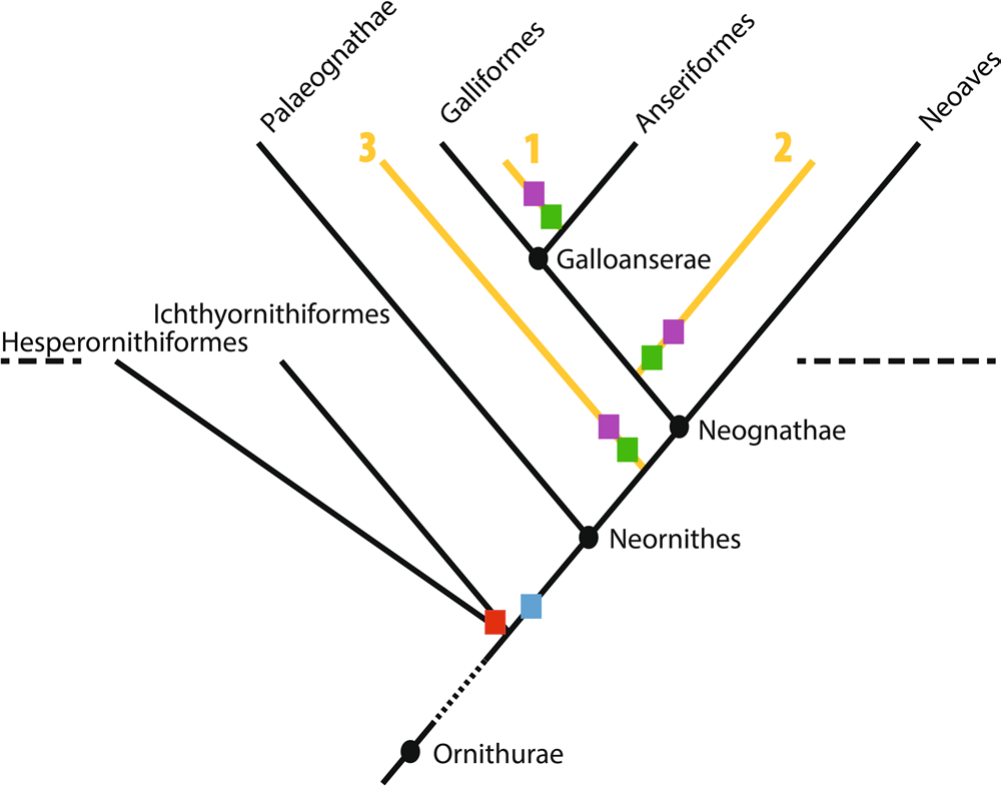
Hypotheses for the phylogenetic position of pseudotoothed birds (Odontopterygiformes). The three hypotheses of phylogenetic placement of the Odontopterygiformes are shown in orange: basal within Anseriformes (1), sister to crown Galloanserae (2), or sister to crown Neognathae (3). Squares indicate the probable position of main innovations related to jaws: acquisition of streptognathism and loss of mandibular symphysis (red square in Hesperornithiformes and Ichthyornithiformes, violet squares in Odontopterygiformes), loss of teeth on the line to Neornithes (blue square), and acquisition of pseudoteeth in Odontopterygiformes (green squares). The horizontal dashed line indicates approximate position of the Cretaceous-Paleogene extinction event.

All living birds are edentulous. Fossil representatives of crown group birds (Neornithes) with documented jaw elements indicate that tooth loss occurred between 125 and 66 million years ago (Ma)^1,2^, whereas genomic data point to 116 Ma^3^. However, members of an extinct Neornithine clade known from almost 60 Ma to ca. 2.5 Ma, the order Odontopterygiformes, large pelagic birds, grew a series of pseudoteeth along their jaw tomia, the cores of which were made of bone^4,5^ (Fig. 1). The earliest Paleocene Odontopterygiformes already had a pseudodentition^6^. Histologically, the bony cores of these protuberances were a continuity of the supporting jaw, as they consisted of the same kind of bone tissue; therefore, they were merely excrescences of the tomial cortex, with their development starting at the end of the circumferential growth of the jaw bones^5^. Pseudoteeth comprise none of the typical hard tissues that form true teeth (dentin, enamel, cement), and are not inserted in alveoli (sockets) -or grooves. Moreover, the occurrence of numerous neurovascular foramina openings at the surface of the pseudoteeth bony cores (Fig. 1) indicates that the latter must have been covered by epithelial sheaths that molded onto their surface^4,5^. These epithelial sheaths are likely to have been keratinized, like the current avian rhamphotheca, albeit relatively late in post-hatching development, suggesting altriciality^5^. The pseudodentition of Odontopterygiformes, strikingly resembles a vertebrate marginal dentition in terms of external morphology (conical, “caniniform” shape), location on jaw tomia, and regular, serial (meristic) distribution. In addition, pseudodentition is characterized by a very peculiar, serial distribution of the pseudoteeth (Fig. 1), alternating by size along the jaw tomia, and involving three to five size classes (“ranks”) in adults, depending on species^4,5,7^ (see also SFig. 1). Here we have ranked the pseudoteeth from 1 (PT1s, the largest) to 4 and 5 (PT5s, the smallest, existing in at least one species; SFig. 2). These alternations are reminiscent of the “waves” of odontogenetic developments described in reptiles^8–11^. The phylogenetic position of pseudotoothed birds among basal Neornithes is still debated, with the most prominent hypotheses being a sister relationship with either crown Anseriformes^5,12,13^, or crown Galloanserae, or crown Neognathae^13^ (Fig. 2).

Until now, the evolutionary and developmental origins of pseudoteeth have been challenging. Nevertheless, by considering the unique characteristics of the pseudodentition, the growth pattern of individual pseudoteeth in one species^5^, as well as new, original lines of evidence, we propose an integrative developmental model which suggests that a diverted odontogenetic potential was probably active (see also Supplementary Text, SText1) in the developmental control of pseudodentition (see also SText 2).

## Late Growth and Rhamphotheca Keratinization

The micro-anatomical and histological characteristics of pseudotooth bony cores in the odontopterygiform *Pelagornis mauretanicus* indicate that their growth on the jaw tomia started during, or shortly after, completion of the circumferential (sub-periosteal) growth of the supporting jaw bones (i.e. growth in diameter or thickness of the dentaries, premaxillaries, or maxillaries)^5^ (see also SText 3). Such a late pseudotooth growth would have been associated with delayed keratinization of the overlying epithelial layers long after hatching^5^ and just prior to independence from the parents, presumably approaching 18 months after hatching in large pseudotoothed bird species (SText 4). Therefore, until this late keratinization, the whole epithelium would have remained alive and continued odontogenetic signaling until late development. In contrast, early epithelium keratinization (e.g. *Gallus gallus*) implies the death of epithelial cells across most of the epithelial depth, rendering interaction with the underlying mesenchyme impossible. Such delayed rhamphothecal keratinization might be paralleled by the existence of a soft rhamphotheca, at least locally, in adult representatives of three extant bird taxa: Anseriformes, Apterygidae and some Charadriidae, lineages that have been toothless for a long time^5^. In the hypothesis of a sister relationship between the orders Odontopterygiformes and Anseriformes (superorder Odontoanserae)^12^, a shared phylogenetic background might have favoured the effects of a mutation which caused delayed epithelial keratinization in pseudotoothed birds. Late epithelial keratinization would be viable for young birds if the latter did not use their fragile, acute, growing pseudoteeth with soft epithelium, but were fed by adults during a prolonged period, at least until the end of pseudodentition growth and full rhamphothecal keratinization^5^.

Comparable altriciality is a derived condition in several lineages^14^; however, altriciality in Odontopterygiformes − be it sister to the Neognathae, the Galloanserae or the Anseriformes − would have been the most basally derived altriciality, given that all basal Neornithes (Palaeognathae, Anseriformes, and Galliformes) are precocial to superprecocial^14^. Delayed epithelial keratinization, a condition of prolonged epithelial signaling in pseudotoothed birds, would have been a non-counter-selected by-product of their derived altriciality.

In addition to this potential role for prolonged epithelial signaling, the exclusive location of pseudoteeth on the tomial edges of the jaws, their individualization, and regular spacing that constituted a serial meristic trait, and, to a lesser extent, their simple, almost conical tooth-like shape, suggest that some developmental bases were shared with typical odontogenesis (see also SText 5). We propose that, near the end of the circumferential growth of supporting jaw bones, delayed oral epithelial keratinization allowed basal epithelial cells to continue expressing odontogenetic signaling molecules, to which competent ectomesenchymal cells, such as in extant toothed vertebrates, would have been able to respond. Additionally, we postulate that in the absence of such odontogenetic ectomesenchymal competent cells – a situation which occurs in extant birds^15^ the osteoblasts of the jaw bone periosteum did respond; enabling oral epithelium-osteoblast interactions to ensue, which induced the differentiation, growth, and shaping of the pseudoteeth. This scenario raises two issues: (i) the effective continuation of epithelial signaling, and (ii) the possibility for periosteal osteoblasts to interact with the signaling basal epithelial cells.

## Efficient Signaling and Responding Osteoblasts

The possible causes of the loss of capability to form teeth in the neornithine basal lineage are as follows: (i) absence of odontogenetic gene expression, among which the expression of *BMP4* by the oral epithelium is a good candidate^15,16^, (ii) lack of competent ectomesenchymal cells which normally migrate from the neural crests into the oral mesenchyme and differentiate into odontoblasts^17^, (iii) lateral shift of the boundary between the epithelium and the mesenchyme^15^, (iv) diversion of gene function toward the formation of a rhamphotheca and subsequent impossibility for the keratinized epithelium to interact with the mesenchyme^1,2^ these possible causes are not mutually exclusive^2^. Nevertheless, the earliest steps of odontogenesis still occur in the chick embryo but the sequence of development stops at embryonic day E5, before the tooth bud stage has been reached. In the *talpid*^2^ (*ta*^2^) mutant chicken embryo, dental development continues further with the production of tooth rudiments; however, as this mutation is lethal, this development cannot continue later than E17^15^. These data indicate that odontogenetic epithelial signaling is maintained and can be reactivated in the chicken upon interaction with competent odontoblasts to form tooth rudiments, as demonstrated by using experimental recombination of chick oral epithelial cells and mouse neural crest-derived cells^17^. We postulate that, in the earliest derived toothless odontopterygiforms (after 50 Myrs length of time, or less, since the ancestral neornithine loss of teeth; Fig. 2) the odontogenetic epithelial signaling was still unaltered: *BMP4* signaling was not inactivated and the basal oral epithelial cells were capable of interacting with mesenchyme-derived cells. Therefore, as teeth did not develop, we assume that competent neural crest-derived ectomesenchymal cells were lacking in the oral mesenchyme, and that odontoblasts could not differentiate in response to epithelial cell signaling. Although the first population of cells originating from the neural crest did presumably contribute to the formation of jaw bones through epithelium-mesenchyme interaction, as in extant birds, the second population of neural crest-derived cells did not differentiate into odontoblasts, contrary to what occurs in toothed taxa^18^. This situation could result from two distinct causes: ectomesenchymal cells did not reach the appropriate jaw region to be able to respond to epithelium signaling, or they were not located in the right place or at the right time (like in extant birds)^17^. Indeed, if these cells had been present, pseudoteeth would have displayed most of the characteristics of true teeth − such as a dentin-like tissue surrounding a pulp cavity and other features − which is not the case. The only alternative hypothesis is that osteoblasts from the jaw periosteum facing the oral epithelium did respond to signaling molecules expressed by the basal oral epithelial cells. This means that periosteal osteoblasts were competent to start such an interaction, and continued until the formation of odontoid excrescences. Indeed, osteoblasts are known to have close evolutionary relationships with odontoblasts^19,20^. Osteoblasts − cells of mesenchymal origin − have strong developmental affinities with odontoblasts of ectomesenchymal origin, as evidenced by numerous examples of toothed vertebrates blurring the limit between bone and dentine with a histological continuum^19^. Alveolar bone first appeared at least in basal amniotes^21^, being identified in a close relative of the last common ancestor of mammals and sauropsids. Hence, both alveolar bone and dentin were present in the Ichthyornithiformes and the Hesperornithiformes, which are the nearest toothed relatives of the Neornithes^22^, and pseudotoothed birds. Both alveolar bone and dentin arise from the same cell population that differentiates into odontoblasts and osteoblasts, respectively; a differentiation process regulated by various genes including *Runx2*^20^. Therefore, we postulate that the periosteal osteoblasts that formed pseudoteeth were capable of responding, with their own potential, to oral epithelial cell signaling.

Another necessary condition for pseudotooth formation in Odontopterygiformes (see SText 6) is that the oral epithelium signaling, and its subsequent interactions with the mesenchyme, continued sufficiently late during ontogeny, ending only when the delayed keratinization of the basal epithelial cells occurred. Indeed, during this period, the jaw bones increased in circumference, which allowed periosteal osteoblasts to interact with the basal epithelial cells due to their close proximity. This necessary time window was made possible relatively late in ontogeny, but nevertheless early enough thanks to prolonged oral epithelium signaling. Based on the known distance of diffusion of the oral epithelial odontogenetic signaling through the mesenchyme in extant toothed taxa, the optimal distance favouring interactions between the basal epithelial cells and the periosteum should be as small as a few tens of micrometers (max. ~100 µm)^23,24^.

## Origin of Shape

**Figure 3:**
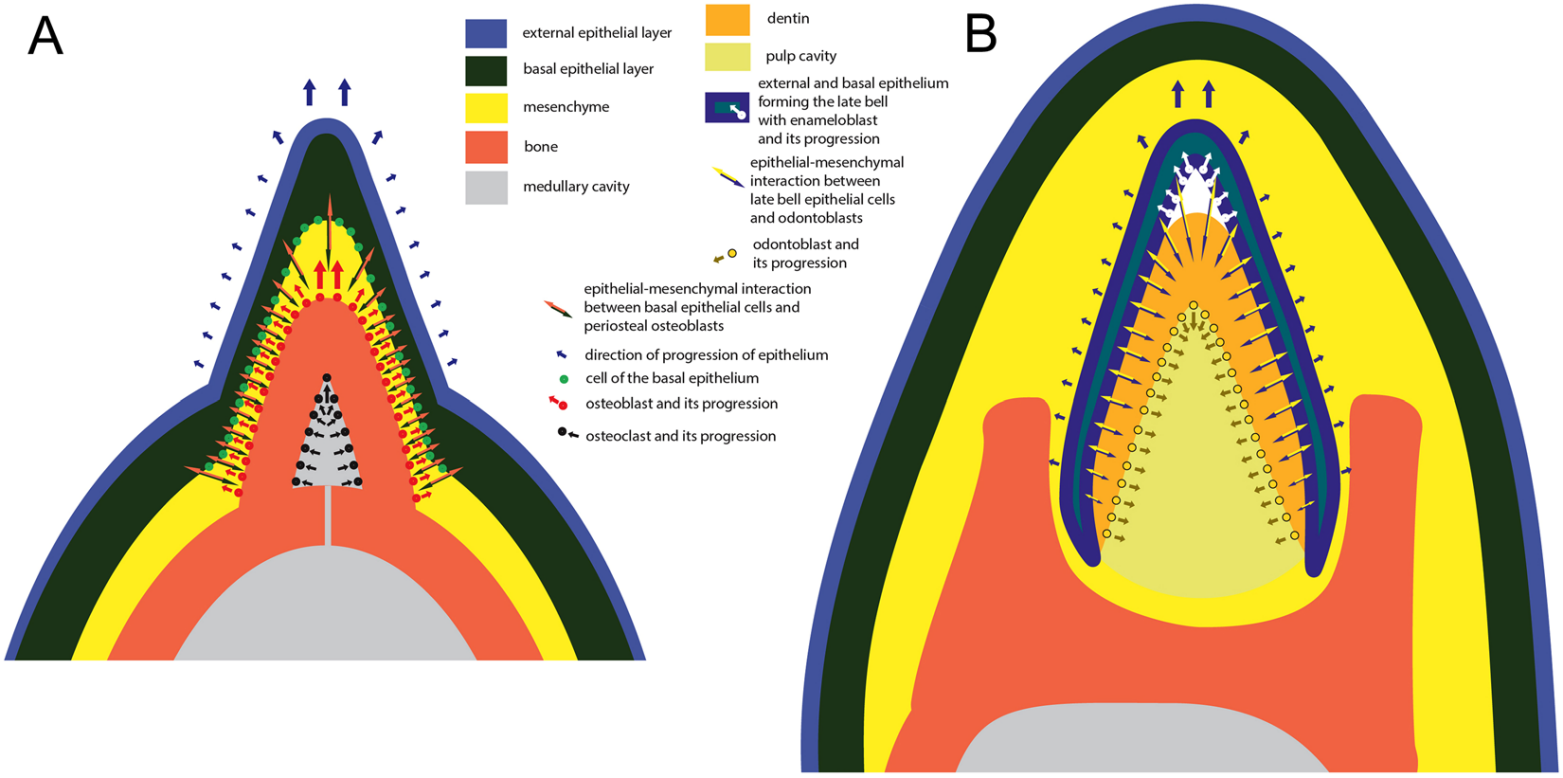
Proposed model of pseudotooth development compared with that of a tooth. Schematized transverse jaw section across a pseudotooth (**A**) and across a true tooth (**B**). In (**A**) the developing pseudotooth is shown at a growth stage approximately equivalent to that of stage 6 in Fig. 4. In (**B**) the developing tooth is shown schematically at “late bell” stage. In both instances epithelial-mesenchymal interactions are indicated, with the different categories of involved tissues, and their direction of growth. Thickness of tissues is exaggerated for convenience of visibility, especially for the mesenchyme.

**Figure 4:**
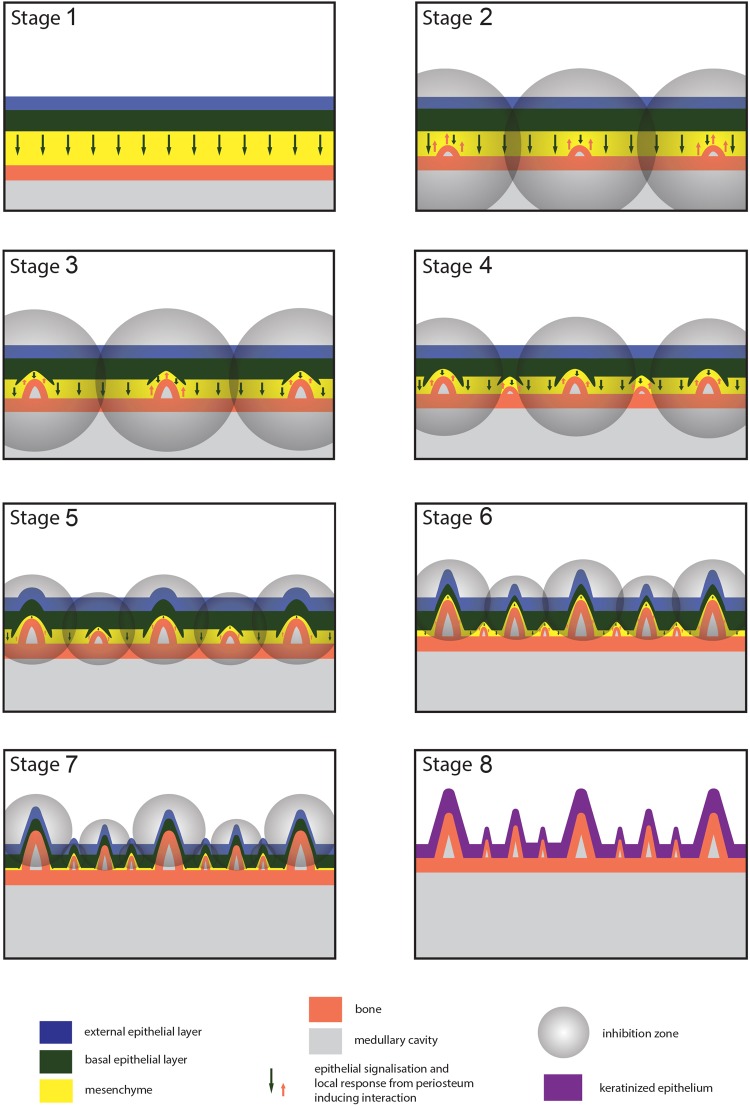
Proposed model of pseudodentition development. The differential growth of the differently ranked pseudoteeth is shown in eight stages, in the parasagittal section of a jaw for all pseudoteeth. For convenience of visibility, tissue thicknesses are exaggerated, ~10 × (bone, epithelium) and ~100 × (mesenchyme). Epithelial-mesenchymal interactions between the basal epithelium cells and the periosteal osteoblasts start when the intervening space becomes lower than ~100 µm (Stage 2). This interaction ends when epithelial cells become keratinized (Stage 8).

Once periosteal osteoblasts had responded to the signaling molecules secreted by the basal cells of the oral epithelium, the genetical cascade controlling epithelial-mesenchymal interactions was initiated, as for normal odontogenesis^25^. Osteoblasts proliferated as meristically individualized populations developing at regularly spaced intervals along the tomial surface of the jaw bones (Figs 3 and 4). Opposite these “pseudotooth primordia”, the basal epithelial cells also proliferated and differentiated into “pre-ameloblasts”, which then developed into dental organs and invaginated as during true tooth development, forming serial bell-like structures. Simultaneously, underneath these, the osteoblasts formed bony cones, the shape of which was controlled by the dental organs (Figs 3 and 4). The conical, caniniform shape of pseudoteeth demonstrates such an “ameloblast”-osteoblast interaction controlling their growth. As the bony cones of the pseudoteeth grew, the dental organs extended, protruding along the external, tomial side. The forming pseudoteeth continued growing until the epithelium started keratinizing and no longer interacting with the osteoblasts, which ceased proliferation and bone formation. The final shape of the pseudoteeth is unicuspid, sub-conical and sharp, though rostro-caudally flattened in lower-ranked (smaller) pseudoteeth (see below), with slight additional features for some of them (ridge, curvature; see STable 1). This process agrees with a general inheritance of the “bell” shape that was presumably present in the closest (and latest) toothed ancestor, judging from the pseudoconical, “caniniform” tooth shape in the sister-taxa of Neornithes, *Ichthyornis* and Hesperornithiformes (notwithstanding the curvature of their rostralmost teeth)^22,26^. It is noteworthy that the “ameloblasts”, although controlling the pseudotooth shape, were unable to deposit enamel matrix proteins on the bone surface. Indeed, all the specific genes encoding these proteins were already invalidated in the earliest Odontopterygiformes, about 50 myrs after the common ancestor of modern birds lost the capability to develop teeth – as they are invalidated in extant birds^27,28^.

The growth of pseudodentition ended after it expressed its unique, alternate distribution of unequally-sized pseudoteeth. The setting of this particular and regular size distributional pattern implies further characterization of our model.

## Origin of Positions

The regular spacing of adjacent PT1s reflects the regulation of a meristic trait. Since the spacing and tomial location of pseudoteeth are identical to those with a true dentition, it suggests a similar process in the setting up of the sites where these meristic structures developed. As hypothesized by several authors for reptilian teeth^9,11,29–31^, the setting up of a serial distribution of the invaginations, where interaction developed, was presumably induced by equally dimensioned and equally-spaced inhibition zones. These morphogenetic fields inhibited the development of another pseudotooth within the radius of a sphere-like zone of influence (Fig. 4). Inhibition is hypothesized to have been induced by the action of molecules diffusing from the initial formation centre of each pseudotooth (around the pseudotooth apex), with all inhibition zones being with the same radius of influence. The molecular evidence of such initial inhibitory zones in dentition development, appearing simultaneously with the initially developing tooth germs, has been presented for trout dentition^32^. In mammals, the involved signaling molecules, including *SHH*, *BMP4*, and *FGF8*, are secreted from the epithelium, resulting in a signaling response from the mesenchyme, which in turn induces the odontogenetic potential of the epithelium, prompting the continuing cascade of epithelial-mesenchymal interactions^17^. In birds, a loss of seriality, concomitant with a loss of dentition and the emergence of a continuous rhamphotheca^2^, may correspond to a change in the *SHH* expression, which becomes more broadly expressed in an antero-posterior direction without a gap^33^. The *SHH* expression in Odontopterygiformes might have retained a serial pattern inherited from toothed ancestors, or re-acquired it independently, this regulating gene being otherwise involved in numerous pathways distinct from odontogenesis.

## Inhibition Zones and Size Distribution

Aside from pseudoteeth shape, location, and meristic characteristics − features homologous to a majority of vertebrate dentitions − adult odontopterygiforms display three to five size classes of pseudoteeth arranged in a regular, sequential manner: PT2s are located midway between two consecutive PT1s, PT3s are located between PT1s and PT2s, PT4s (if present, depending on species) are situated between PT3s and PT2s and between PT3s and PT1s (Fig. 1, STable 1); tiny intervening PT5s were observed here in only one case (SFig. 2).

The hypothetical causes of the sequential size distribution of equally-spaced pseudoteeth, which can be formalized as a series of pseudoteeth, PT1-(5)-(4)-(5)-3-(5)-(4)-(5)-2-(5)-(4)-(5)-3-(5)-(4)-(5)-1 (Fig. 1), can be classified as four (not strictly exclusive) modes of growth: (i) synchronous start of the growth of PTs, of all ranks, and their differential growth rates; (ii) synchronous start and asynchronous end; (iii) asynchronous start and synchronous end. A last hypothesis (iv) would be that an asynchronous start of growth (later start for smaller pseudoteeth) would indirectly derive from a simultaneous elongation of supporting jaw bones, resulting in an increase of the distance between two adjacent large PTs, and providing a new space with minimal growth inhibition midway between these. However, there is no evidence of an increase in intervals when comparing juvenile odontopterygiform fossils with adults (SText7); therefore hypothesis (iv) can be discarded. Indeed, just prior to pseudotooth growth, the circumference of the jaw bones was fully grown, and any elongation could only have occurred at their rostral and caudal ends. Hypothesis (i) − involving different growth speeds in pseudoteeth of different ranks − has also been dismissed, as no such differences can be inferred from paleohistology^5^. Juvenile fossil jaws show faint bumps at the location of the future, adult pseudoteeth of lower ranks (see SText 7; SFig. 2), indicating that the latter started developing much later than higher-ranked (larger) ones. This observation supports hypothesis (iii), i.e. asynchronous start of growth and synchronous end, as opposed to hypothesis (ii).

In the framework of an asynchronous start and a synchronous end of pseudoteeth growth, we propose that the dynamic action of inhibition zones controlled the differential starts of growth, and therefore the final relative sizes of the differently ranked pseudoteeth.

Once inhibition zones were established with the first developing PT1s, determining seriality, they continued to control and limit the growth of adjacent pseudoteeth. This dynamic process caused the inhibition zones to decrease in radius through developmental time, as proposed earlier for reptilian tooth formation^11,29,30^. Likewise, but on a smaller scale, inhibition zones among cusps in a single tooth are known to originate from enamel knots, in some mammals, and to contribute to the regulation of the development of adjacent, secondary enamel knots of ultimately smaller cusps^34^.

As soon as the almost conical pseudoteeth started to grow, zones of inhibition would have set up in the form of spheres of molecular influence, centered on the apex (equivalent to the enamel knot of developing vertebrate teeth^25,35^) of the invaginating basal epithelial layer. These putatively sphere-like zones of inhibition prevented the development of adjacent pseudoteeth within their field of influence. While the first series of pseudoteeth were developing (future PT1s), their respective inhibition zones were progressively decreasing in radius. As a consequence, spaces became free from inhibition at locations equidistant from two consecutive PT1s. The PT2 series could then start developing through epithelial-osteoblast interactions, which in turn produced their own smaller inhibition zone (Fig. 4). This process could also occur later in development so that PT3s could start growing between PT1s and PT2s (Fig. 4), inducing their own, even smaller inhibition zone. Further on, a fourth (and fifth) series could start growing in species bearing four-ranked (and five-ranked) pseudodentitions. Pseudoteeth emerged progressively (largest first) as excrescences of the tomia, formed by bone and covered by epithelium. The final developmental stage of the pseudodentition saw each pseudotooth attain its definitive size, presumably consecutive to rapid epithelial keratinization. This fast but late epithelial keratinization might have been genetically programmed; alternatively, we suggest that it could have resulted from an epigenetic cause. The start of mechanically using the beak tomia by the young at the end of the altricial period would have initiated the keratinization process − local mechanical stress is known to induce keratinization^36^ − which would have proceeded from the external to the basal layer of the oral epithelium, finally stopping interaction and pseudotooth growth.

Most differences in the size distribution of pseudoteeth across various odontopterygiform species (or unnamed specimens) can be interpreted in terms of the species-specific characteristics of inhibition zones, and their modifications during development (SText 8, STable 1). Nevertheless, a consistent correlation emerges and adds support to our model. In species with a larger interval between PT1s (and/or smaller PT1s), the PT3s show an attenuated size decrease relative to PT2s, compared with the size decrease of PT2s vs PT1s (SText 8). This is what would be expected if the inhibitory action of the larger pseudoteeth actually delayed the growth of the last emerging (lower-ranked/smaller) pseudoteeth − the latter reaching a size limited by the duration of inhibition by the larger pseudoteeth through pseudodentition growth. This duration of inhibition, in our model, is greater with larger PT1s and/or with a smaller interval between them. Another characteristic, common to all taxa, is that the lower-ranked pseudoteeth are increasingly more constricted rostro-caudally. This strongly suggests that increasingly constraining inhibition zones during the development of the lower-ranked pseudoteeth, with a smaller intervening space, imposed narrower spaces for rostro-caudal development. This can be clearly seen in the *Pelagornis* species, where the PT4s are blade-like (when present) (SText 9; SFigs 3 and 4). Aside from the normal features of pseudodentitions and interspecific differences, irregularities often occurred within particular specimens. The causes of these irregularities are often blurred by other sources of variation, such as consistently wider spacings, and larger pseudoteeth at the mid-length of the jaw rather than towards the rostral or caudal extremities (SText 10). In certain circumstances, however, morphogenetic interpretations can be proposed; for instance, in the comparison of the right and left jaw areas of a single specimen, at the same level, with one side differing from the other in terms of spacing. Such an example shows that a larger space between two pseudoteeth corresponds to a larger intervening pseudotooth (SText 11; SFig. 4) and provides some support to our hypothesis of dynamic inhibition zones, where a wider space allowed either more numerous or larger pseudoteeth.

The particular sequential size distribution of pseudoteeth is paralleled by that of teeth in the dentition of several fish lineages (e.g. in the jaw dentition of the characiforms Cynodontidae and Erythrinidae, and the teeth of the ‘saw’ in the sawsharks of the genus *Pristiophorus*; SFigs 5 and 6). These similarities in size distribution, between these dentitions and pseudodentitions, suggest that they shared a developmental mechanism of differential growth regulation via inhibition zones; a comparison that holds with the setting up of the first dentition in *Pristiophorus* (though not during its subsequent tooth replacements)^37,38^. In the longnose sawshark, *P. cirratus*, the largest teeth grow first, followed by the medium-sized ones, and finally the smaller ones^37–39^, which is similar to the growth sequence considered in our model for pseudodentition. These observations add further support to our hypothesis, since they attest to a possible common underlying mechanism operating on true teeth as well as on pseudoteeth.

## Pseudodentition Deep Homology with Dentition, and Co-Adaptation with Unique Mandibular Characters

Pseudoteeth differ from true teeth, not only in the tissues involved (bone and keratinizing epithelium vs dentin and enamel) and the lack of pulp cavity, but also in their growth and mineralization dynamics, and in relation to the jaw bone. In true teeth, dental tissues develop and mineralize centripetally before attachment to the jaw bone, and once their final shape is achieved the covering tissue deposited by the ameloblasts (enamel) is highly mineralized prior to eruption. In contrast, the bony cores of pseudoteeth were already part of the jaw bone, and they developed and mineralized centrifugally, through a combined process of external accretion and internal resorption of the cortex. The epithelium covering the bony cores of the pseudoteeth became hardened by means of keratinization (like the rest of the rhamphotheca) after ‘eruption’ (emergence) and completed growth. Therefore, in the model we propose, the only common process when comparing pseudotooth and true tooth formation consists of the epithelial-mesenchymal interactions modeling the final shape, and the capability of osteoblasts to respond to epithelial signaling, as odontoblasts do for teeth. This behaviour is made possible by (i) the common evolutionary origin of osteoblasts and odontoblasts, and (ii) the close vicinity of the interacting cells, osteoblasts, and basal layer epithelial cells. This genetic cascade regulates relatively simple shape, differential growth, and seriality along the tomia. Such an opportunistic diversion of available epithelial odontogenetic signaling making the regulation of bone growth possible is unique among vertebrates. This attests to the considerable plasticity of a developmental program, which is capable in forming highly derived structures similar to dentition, despite the loss of ability to develop true teeth, in crown group birds. As such, this evolutionary innovation not only produced organs that were functionally convergent with dentitions, but is also a new illustration of a kind of deep homology^40^. Future epithelium-mesenchyme recombination experiments might be a mean of testing the model presented here, but previous attempts were not conclusive, due in particular to extremely difficult separation between these two tissues^41^. On the other hand, current knowledge on genic cascades implied in tooth initiation in mice^42^ makes it possible to envision experiments involving beads, impregnated with corresponding epithelium-mesenchyme signaling molecules. These beads would be implanted (*in vivo* or *ex vivo*) between chick embryo oral epithelial and mesenchymal tissues.

Once the process of pseudodentition growth was acquired by an ancestral odontopterygiform, it was maintained in derived lineages presumably for adaptive reasons (see also SText 12). Among Ornithurae, the combination of streptognathism and absence of mandibular symphysis only occurs in the Odontopterygiformes and the Cretaceous toothed avian taxa Ichthyornithiformes and Hesperornithiformes, the respective sister taxa of crown group birds^5,43^. This conformation weakened grasping strength^5,43^ and, in the absence of teeth in odontopterygiforms, pseudodentition could have acted as a counterbalancing grasping enhancer. The conformation of the mandible would have evolved in co-adaptation with the pseudodentition. This evolutionary resourcefulness produced structures possibly less resistant than teeth^44^. Nevertheless, pseudotoothed birds were extremely successful, wandering the world’s oceans for more than 55 million years^6,7^.

## Methods

### Observations and X-ray microtomography

Specimens of *Dasornis* (Paleocene-Eocene, Morocco)^6^ and *Pelagornis* (Pliocene, Morocco)^5^ were examined for comparison, as well as specimens of *Hesperornis regalis* (late Cretaceous, North America)^22^. The *Dasornis* and *Pelagornis* fossils were imaged using conventional X-ray microtomography, performed at the Ecole Normale Supérieure de Lyon and at General Electrics (Lyon) using Phoenix Nanotom. The *Hesperornis* fossil was imaged using synchrotron X-ray microtomography at the European Synchrotron Radiation Facility (Grenoble). The 3D volumes obtained were analyzed using VGStudio MAX 2, as previously described^5,22^.

## Electronic supplementary material

Supplementary Information

## Data Availability

Data analysed during this study are included as Supplementary Information files. Any additional data are available from the author upon reasonable request.
