# Supplementary material for: Bony pseudoteeth of extinct pelagic birds (Aves, Odontopterygiformes) formed through a response of bone cells to tooth-specific epithelial signals under unique conditions
Source: Sci Rep. 2018 Aug 28;8:12952. doi: 10.1038/s41598-018-31022-3 (PMC6113277; doi:10.1038/s41598-018-31022-3)
Supplement: Supplementary file 1 — Supplementary Information [file 41598_2018_31022_MOESM1_ESM.pdf]

**Supplementary Information accompanying Louchart et al. "**Bony pseudoteeth of extinct pelagic birds (Aves, Odontopterygiformes) formed through a response of bone cells to tooth-specific epithelial signals under unique conditions"

Antoine Louchart, Vivian de Buffrénil, Estelle Bourdon, Maïtena Dumont, Laurent Viriot and Jean-Yves Sire

## Supplementary text

**Supplementary Discussion, section 1. Alternative developmental models would not be consistent with data.**

The only alternative developmental model incorporating elements of analogy or homology with support from extant organisms (other than the model we propose in the main text), would be the development of spurs on the legs of some galliform birds and cassowaries[1], with additional examples of similar spurs on the carpometacarpi or metacarpi in other diverse avian taxa[1-3]. The development of the tarsometatarsal (leg) spurs has already been investigated[4-6], and like pseudoteeth these spurs consist of pseudoconical bony cores covered with a keratinous sheath, which is acute in several species. In a few species, these leg spurs have a meristic distribution, i.e. several spurs are present on each tarsometatarsus and are evenly spaced along the tarsometatarsus shaft[7]. Developmentally, however, the keratinous sheaths of the leg spurs grow and become hardened before the spurs bony cores form. The condensation of the spur's bony core starts just as the bone separates from the tarsometatarsus, inside the forming spur sheath, near its apex – away from the almost fully grown tarsometatarsus shaft[4,5,8]. The osteoblasts located at the base of the spur bone then interact with the periosteal osteoblasts at the surface of the tarsometatarsal shaft, and both surfaces (spur bone base and adjacent tarsometatarsus surface) join together through limited osteoblast proliferation[4-6,8,9]. Spur bone is secondarily fused to the tarsometatarsus shaft surface; a process which leaves obvious marks at their junction[4,5,8]. Therefore, spur and tarsometatarsus bones initially develop from separate condensations and grow independently until an advanced stage[4,5,8,10]. In contrast, the bony cores of pseudoteeth are excrescences of the jaw bone periosteum by means of osteoblast proliferation; a process which is activated through interactions with the basal layer of the epithelial cells that are not yet keratinized.[11]

**Supplementary Discussion, section 2. Pseudodentition is characterized by the unique size distribution and acute shape of pseudoteeth, compared with bony "odontoids" in other vertebrates.**

Bony odontoids in place of teeth constitute a rare feature in vertebrates. In birds, apart from the Odontopterygiformes, they are known only in the extinct insular moa-nalo (flightless giant ducks from the Hawaiian Islands) of the genera *Thambetochen* and *Ptaiochen*[12]. Moa-nalo odontoids resemble the equally-sized thick lamellae of extant geese rhamphothecae. In several anuran lineages, bony odontoids range from fangs to paired bumps[13,14]. The teleost fish *Danionella dracula* exhibits bony fangs and a series of small odontoids[15]. Bony odontoids are also known from several chelonian lineages[16]. The single, symphyseal bony odontoid described in the mandible of an istiodactyl pterosaur, also called a pseudotooth[17], does not actually resemble the odontopterygiform pseudoteeth, the latter forming paired series in both mandible and rostrum. None of these examples resemble the large acute odontopterygiform pseudoteeth, which resemble teeth of a jaw's normal dentition and exhibit a particular, regular and sequential size distribution. Therefore, we have restricted the use of the terms

pseudotooth and pseudodentition to the Odontopterygiformes. Differences in shape between the bony pseudodentition and its horny cover (i.e. the rhamphotheca covering the jaw bone) might have existed, due to putative variations in rhamphothecal thickness (unknown because it is not preserved during fossilization) according to the size of the pseudotooth bony core covered. The larger pseudoteeth (PT1s and PT2s) had an acute rhamphothecal cover (at least as acute as their bony cores), as shown by the corresponding holes for mandibular PT1s and PT2s, located on the ventral side of the bony rostrum in sufficiently complete specimens[18,19]. Mandibular PT1s and PT2s interlocked into these rostral holes when the jaws were closed. Based on observations on extant large-beaked birds[20], rhamphothecal thickness in pseudotoothed birds probably ranged from 0.5 mm (over most of the jaw) to a few millimetres in certain regions (e.g. tomial edges, beak tip), perhaps 1-2 mm over the pseudotooth bony cores. Many other birds show serrations on the beak tomia, but these serrations are obtuse and reduced in size – a few millimetres at the most in geese; they are equally sized along the jaws, and exclusively affect the rhamphotheca rather than the underlying bone[1,21].

**Supplementary Discussion, section 3. Ontogenetic differences between "tomial teeth" and pseudoteeth.**

The two paired "tomial teeth" reported in the cranial extremity of the rostrum in *Pelagornis orri*[22] and *P. chilensis*[19,23] are extremely similar, and perhaps homologous to the two paired tomial teeth of the extant *Harpagus bidentatus* (Accipitridae; phylogenetically distant from pseudotoothed birds) (SFig. 1). These structures are (i) located exclusively at the rostral (anterior) end of the rostrum, (ii) obtuse and rounded in outline, and (iii) latero-medially flattened unlike pseudoteeth. Tomia are generally blade-shaped like tomial teeth, whereas pseudoteeth, with their much wider base, require a latero-medial widening of the underlying jaw tomia. Tomial teeth are not homologous to pseudoteeth, but they might deserve further investigation with respect to a wider category of "odontoids" in birds. In contrast to pseudoteeth, "tomial teeth" presumably grow synchronously with the main jaw bone. In *P. sandersi* only a single paired rostral tomial tooth is reported[24], but the above interpretation also applies to that species. Several extant avian taxa aside from *H. bidentatus* exhibit double or simple paired tomial teeth[21].

**Supplementary Discussion, section 4. Estimation of the post-hatching growth timing of the beak and pseudodentition in pseudotoothed birds.**

Extant albatrosses (Procellariiformes, Diomedeidae) provide interesting elements for estimating the plausible timing of beak growth in pseudotoothed birds, because (i) both taxa share morphological (skeletal) characteristics corresponding to similar adaptations for pelagic foraging, (ii) pseudotoothed birds were most probably altricial in development like albatrosses, and (iii) albatrosses are the largest pelagic seabirds, approaching medium species of Odontopterygiformes in size. In the albatross *Diomedea chrysostoma* (2.20 m wingspan), the definitive beak (culmen) length is reached ca. 100-110 days after hatching[25]. In the same species, fledging (independence of young from parents, which means first use of pseudodentition for catching prey) occurs at 140-150 days after hatching. In the smaller *Calonectris diomedea*, a shearwater (Procellariiformes, Procellariidae; 1.20 m wingspan), adult beak (culmen) length is reached at ca. 60 days, and the adult beak height earlier (like in "long" beaked birds in general) at ca. 35-40 days. Fledging occurs at 95 days (on average) after hatching[26]. Hence, in the shearwater there is a period of ca. 60 days between the end of beak growth in height (i.e. circumference) and independence. Even in the albatross, adult beak height is reached before adult length. There is already a period of 40 days between complete growth in beak length and independence, and perhaps up to 70 days might be inferred between the end of circumferential growth and independence, i.e. ca. 40 % of fledging time.

In the largest albatross *D. exulans* (wingspan over 3 m), fledging occurs at ca. 270 days (nine months) after hatching. Using the relation between its fledging time and body size (and wingspan in these birds) an extrapolation can be attempted in *Pelagornis mauretanicus*, with a wingspan of over 5 m[27], where fledging might have occurred more than one year after hatching, probably around 18 months. If this time span amounted to 40% (like in *D. chrysostoma*), it would roughly indicate that eight months elapsed in this pseudotoothed bird between completion of circumferential growth of beak (i.e. start of pseudoteeth growth) and independence (i.e. end of keratinization of the rhamphotheca, at the end of pseudoteeth growth). Such an estimate for pseudoteeth growth duration is coherent with our growth model and histological observations[11].

**Supplementary Discussion, section 5. Evaluating the pertinence of comparison between pseudoteeth and first generation archosaurian teeth, or *talpid2* chicken teeth rudiments with regards to pseudotooth development.**

The purported, superficial resemblance of pseudoteeth with (i) first generation teeth in archosaurians and (ii) tooth rudiments in *talpid2* chicken mutant embryos[28], has led in the past to suggestions of some level of homology between the development of pseudoteeth and true teeth[19,29,30]. This argumentation, however, is questionable, and not relevant for understanding pseudotooth development. Pseudoteeth differ intrinsically from avian teeth, which are composed of dentin, cement (at least in some taxa), and an enamel crown cover, like most teeth[31], whereas pseudoteeth are composed of bone and a rhamphothecal cover[11,29,32,33] (incidentally, teeth are not “covered by dentine” *contra* Mayr and Rubilar[19]). Nevertheless, it was suggested that tooth-specific developmental programs might be responsible for pseudotooth development, based on their putative resemblance with the early developmental stages of first-generation archosaurian teeth[19]. However, these earliest “surface teeth” stages are not mere outgrowths of the jaw bone (*contra* Mayr and Rubilar[19]), but projections of the epithelium[34], in which mesenchyme-derived odontoblast precursors differentiate and form dentin[35]. These teeth are not made of bone and are situated far from the jaw bone; later generation teeth develop deeper into the mesenchyme, from a dental lamina, and only later elongate to fuse with the jaw bone into alveoli[34,35]. Therefore, there is no complete or direct homology (*contra* Mayr and Rubilar[19] and Mayr and Zvonok[30]) between pseudoteeth, which are bony outgrowths at the jaw bone’s surface, and first-generation teeth in archosaurians (and presumably tooth rudiments in *talpid2* chicken mutants)[28], that are early tooth precursors situated far from the jaw bone with a mesenchymal component made of dentin (predentin in *talpid2* mutant embryos at ca. 17 days)[21]. Therefore, the composition and precise location of pseudoteeth differ radically from those of first generation teeth in archosaurians as well as the tooth rudiments of *talpid2* embryos. The latter two structures do not illustrate any developmental link between pseudoteeth and teeth. In contrast, we have based our model on the general location, seriality and sequential size distribution of pseudoteeth (see Supplementary table STable 1), their shape and growth timing[11], plus several other independent lines of evidence (present study; see Main text).

**Supplementary Discussion, section 6. Other categories of avian odontoids arose independently, even though a common background for Odontoanserae might be at stake.**

The only other birds that possess bony odontoids on the jaws (the extinct Hawaiian moa-nalo of the genera *Thambetochen* and *Ptaiochen*)[12], albeit much less developed and acute than odontopterygiform pseudoteeth and lacking the sequential size distribution of the latter, are Anseriformes (large flightless ducks of the tribe Anatini)[36]. Within the hypothesis of a sister-relationship between Anseriformes and Odontopterygiformes[11,29,37], one could suggest that the

potential of the jaw periosteum to develop bony odontoids might be shared by members of the Odontoanserae, at least. In both moa-nalo and odontopterygiform birds, developmental heterochrony involving jaw growth and rhamphothecal keratinization might be at stake for this potential to be expressed. In the case of moa-nalo, heterochrony related to flightlessness is already assumed for other anatomical parts[38]. A favourable background for the evolution of delayed rhamphothecal keratinization exists in Anseriformes, one of the few bird groups with partly soft rhamphotheca, at least locally, in adults[11].

**Supplementary Discussion, section 7. Juvenile specimens provide clues for the growth sequence of the different ranks of pseudoteeth.**

All but two of the odontopterygiform bird specimens with preserved pseudodentition are adults, based on the non-fibrous external aspect of the bone and on the pointed shape of the unbroken pseudoteeth. The two exceptions consist of: (i) a jaw bone fragment of a juvenile of *Lutetodontopteryx tethyensis* from the Middle Eocene of Ukraine[30], and (ii) a jaw bone fragment of a juvenile of cf. *Pelagornis* sp. from the Early Miocene of Venezuela[23] (SFig. 2). Incidentally, the latter fossil was not recognized as a juvenile by the authors, although this is unambiguous. Both fossils exhibit the well-marked fibrous external aspect of the cortical bone, typical of the osteologically juvenile stage. Whereas the adult specimens of *L. tethyensis* from the same locality as the juvenile exhibit three size ranks of pseudoteeth, the juvenile specimen shows only two (PT1s and PT2s). However, tiny caudo-cranially constricted bumps – as constricted as the smallest pseudoteeth of *Pelagornis* – are clearly visible at locations where the third rank pseudoteeth (PT3s) would be expected to develop further toward the adult stage (see fig. 2R in Mayr and Zvonok[30]; reproduced with modifications in SFig. 2). Most of them are emergent PT3s, and show a strongly cranio-caudally constricted elliptical base shape, as in the PT4s of other taxa (except perhaps the first visible bump on the right side, which seems to be a partly broken base, as seen in some PT4 specimens of *P. mauretanicus*[27]). The presence of only two developed size ranks in this juvenile specimen (even though all pseudoteeth are broken due to their fragile, thin cortex), and PT3s only at the bump stage, reflect incomplete development of the pseudodentition at this individual's time of death of. This indicates that PT3s start developing later than PT1s and PT2s, in this species at least. Similarly, the juvenile mandible of cf. *Pelagornis* sp. from Venezuela exhibits fully grown PT1s, PT2s, and PT3s (albeit broken), whereas PT4s and probable PT5s are only at the bump stage at the individual's time of death (see fig. 2 in Solórzano and Rincón[23]; reproduced with modifications in SFig. 2). To summarize, PT3s start growing after PT1s and PT2s, and PT4s and PT5s (when present) start growing after the three higher ranks of pseudoteeth. Hence, juvenile odontopterygiform specimens provide evidence for a model of an asynchronous start and a synchronous end of growth for the different ranks of pseudoteeth, with PT1s developing first. Incidentally, the juvenile specimen from Venezuela provides the only evidence that some pseudotoothed birds had five ranks of pseudoteeth (with even additional bumps being irregularly present), albeit with PT4s and PT5s at an early growth stage (SFig. 2). The two juvenile specimens also illustrate the rostro-caudal constriction of smaller pseudoteeth, increasing with rank, which is also obvious in adults of most species. Finally, the ratio between PT1s intervals and jaw bone height is not greater in adult odontopterygiform specimens than in juveniles, which indicates that the relative intervals between PT1s do not increase with growth.

**Supplementary Discussion, section 8. Interspecific variability in the distribution of pseudoteeth suggests the existence of species-specific characteristics of inhibition zones.**

Differences exist between odontopterygiform species in terms of the distribution and shape of pseudoteeth: height and relative rostro-caudal width of PT1s, spacing between PT1s, ratio

between PT1 measurements and their intervening spaces, width of pseudoteeth divided by width of immediately higher ranked pseudoteeth, or width of pseudoteeth divided by spacing between adjacent pseudoteeth, among other ratios. Among the three-ranked species, a relation exists between the ratio of the interval separating PT1s divided by PT1 width, and the amplitude of the decrease in width of PT3s vs PT2s compared with PT2s vs PT1s (see Supplementary Text 8 table). In species where the value of the distance separating PT1s divided by PT1s width is larger, the decrease in size in a (n) ranked pseudotooth versus a (n-1) ranked pseudotooth is stable from PT1s to PT3s. In contrast, in species where the value of the intervening space between PT1s divided by PT1s width is smaller, the size reduction increases from PT1s to PT3s, and is greater in PT3s vs PT2s, than in PT2s vs PT1s. This suggests that the intervening space between PT1s (coupled with PT1 width) constrains and restricts the size of smaller (lower-ranked) pseudoteeth, including PT3s, which emerge later in our model. This observation conforms to our model of pseudotooth growth control by inhibition from adjacent, higher-ranked (larger) pseudoteeth (we use the pseudotooth base width as the size proxy, rather than height, as pseudotooth tips are generally broken due to post-mortem fractures).

| Species | **Ratio: PT1 base cranio-caudal width/intervening space between PT1s** | PT2 width/PT1 width; PT3 width/PT2 width | **Ratio (mean values): (PT3 width/PT2 width) / (PT2 width/PT1 width)** |
|---|---|---|---|
| *Dasornis emuinus* from Morocco | **0.18** | 0.5-0.6; 0.5-0.6 | **1.00** |
| *Dasornis toliapicus* from England | **0.17** | 0.7-0.74; 0.6-0.8 | **0.97** |
| *Caspiodontornis kobystanicus* | **0.17** | 0.6; 0.5 | **0.83** |
| *Pelagornis chilensis* | **0.23** | 0.5-0.6; 0.4-0.5 | **0.82** |
| *Lutetodontopteryx tethyensis* | **0.25** | 0.6; 0.4-0.5 | **0.75** |
| *Pelagornis longirostris* | **0.25** | 0.5-0.6; 0.3-0.4 | **0.64** |

**Supplementary Text 8 table.** The three-ranked odontopterygiform species show a relation between a greater PT1 size vs intervening space (left column in bold), and a greater relative decrease in size from PT2 compared to PT1, toward PT3 compared to PT2 (decreasing ratio between these two ratios, in the right column in bold). Species ordered from no decrease to a greater decrease (right column in bold) are generally ordered from a smaller to a greater ratio (PT1 width/intervening space) (left column in bold). When plotted against each other, the values of these two parameters determine a linear regression with a $R^2$ of 0.67; the straight line equation is $y = -2.82x + 1.42$ (in x, ratio: PT1 base cranio-caudal width/intervening space between PT1s; in y, ratio (mean values): (PT3 width/PT2 width)/(PT2 width/PT1 width). NB: four-ranked species values show no difference within these parameters. Data from Supplementary table STable 1.

Other measurements or ratios show no consistent correlations (i) with the presence or absence of PT4s, and (ii) with each other among all species, or among three- to four-ranked species (STable 1). Most species are represented by one or only a few specimens, which might lead to

an overestimation of the interspecific variability in relation to intraspecific variability. Several features still show consistent differences between species, which suggests that species-specific characteristics of inhibition zones and their modifications through development are at stake, such as their strength (radius of action), duration of action, and speed of radius reduction. For instance, the presence of PT4s in certain species (STable 1) might be caused either by a lower radius and/or a faster radius reduction of pseudotooth inhibition zones, or by delayed end of growth (epithelium keratinization).

**Supplementary Discussion, section 9. Variation in pseudotooth shape in relation to position (rank) adds support to the hypothesis of dynamic inhibition zones.**

Beside the pseudoconical (caniniform) shape of pseudoteeth in all odontopterygiform species, some display other, specific characteristics, such as caudally hooked higher-ranked pseudoteeth (e.g. *P. chilensis*), the presence of a strong basal-apical ridge in caudo-lateral position in higher-ranked pseudoteeth (e.g. *P. mauretanicus*, *P. chilensis*), varying degrees of forward (rostral) slanting in higher-ranked pseudoteeth, or varying degrees of pseudotooth robustness (essentially starting with PT1s; sharp vs obtuse) (STable 1). Beyond these features, pseudoteeth of different ranks also differ in terms of rostro-caudal constriction within all odontopterygiform species[11]. In all species of *Pelagornis* and *Lutetodontopteryx*, PT1s are pseudo-conical, and lower-ranked pseudoteeth are increasingly more constricted rostro-caudally (SFigs. 3, 4), with PT4s and even PT5s (in species bearing them) being blade-shaped (see SFig. 2). For instance, in *P. mauretanicus*, the ratios of rostro-caudal width to latero-medial thickness at the base of pseudoteeth range from 1.1 in one PT1, to 0.77 in one PT2, then ~0.65-0.75 in PT3s, and 0.34-0.63 in PT4s[11,27] (SFig. 3). In species of *Dasornis*, the trend is the same, starting with the base of PT1s being rostro-caudally longer than in *Pelagornis*. In the *Dasornis toliapicus* specimens from the Paleogene of Morocco (SFig. 4, STable 1), the ratios of rostro-caudal width to latero-medial thickness at the base of the pseudoteeth range from 2.50-4.00 in PT1s (n=3), to 0.92-2.55 in PT2s (n=3), and 0.76-1.15 (n=5) in PT3s. Consequently, in *Dasornis*, the base of pseudoteeth is longer rostro-caudally than the base of pseudoteeth of the same rank in *Pelagornis*, but all species of both genera share a relative increase in rostro-caudal constriction in increasingly smaller (lower-ranked) pseudoteeth.

Incidentally, in *Pelagornis* spp., *L. tethyensis*, and also the *Dasornis emuinus* specimens from Morocco, the latero-medial thickness of tomia equals the latero-medial basal thickness of PT1s, but the smaller pseudoteeth are thinner at their base and located at the lateralmost edge of tomia, so that all pseudoteeth are aligned laterally but not medially (the most "shifted" laterally being the smallest, PT4s or PT3s[11]; SFig. 3). This suggests that the centres of pseudoteeth development appear at the lateral edge of the tomia. (In the *Dasornis toliapicus* from Morocco, higher-ranked pseudoteeth are relatively thin medio-laterally, so that the tomia are thin as well; therefore, the base of lower-ranked pseudoteeth is barely thinner than the tomia, so that their lateral implantation is not obvious; SFig. 4).

**Supplementary Discussion, section 10. Irregular, abnormal distribution of pseudoteeth within some specimens or individuals.**

Irregularities in the presence/absence, exact position, relative size, and/or spacing of pseudoteeth of various ranks occur in almost all of the species/specimens. For instance, in some cases a small, supplementary pseudotooth the size of a PT4 – even in species lacking these – is intercalated between two pseudoteeth but absent from the rest of the jaw (e.g., *P. orri*; see STable 1). Another example is the occurrence of two "small PT2s" side by side, smaller than expected and just slightly larger than the PT3s intervening between them and the much larger PT1s; this is the case in the large '*Dasornis*' specimen from Ukraine described in Mayr and Zvonok (2011: text-fig. 2 A,B)[39]. For some unknown reason, these two PT2s

emerged side by side in place of a single larger expected PT2, and this indeed resulted in their smaller size. In the frame of our model, their smaller size might be explained by the conjunction of a reciprocal inhibition effect from the PT2s themselves, and greater proximity to PT1s, implying longer inhibition. The jaw elements of each species are represented by single individuals (minimum number of individuals based on these jaw elements; MNI= 1; see STable 1), except for *L. tethyensis*, *Dasornis/Gigantornis* from Ukraine, and *D. toliapicus* from Morocco (MNI: near 2 for each), and *P. orri* (MNI: 4 or 5) (STable 1). Even in cases with an MNI above 1, no given part of the jaw can be compared with a homologous part from another individual, due to poor preservation or lack thereof. Therefore, specimens with irregularities stand as isolated cases, and are not easily interpretable in terms of their morphogenetic causes, since several parameters might be involved. For instance, there is the "normal" modification along the jaw (rostro-caudally) in terms of pseudotooth sizes and intervals, in all specimens, and which confounds the "accidental" variations: namely the spacing between pseudoteeth and their size decrease toward the cranial and caudal ends of jaw bones. Incidentally, the latter pattern could be explained by smaller inhibition zones at the jaw's extremities, because these jaw regions developed later and closer to the end of pseudoteeth growth. Some variations might also be age-related (especially given our hypothesis of the late development of pseudoteeth relative to the rest of the jaw skeleton). Pathology or bone reconstruction following fractures cannot be excluded in some cases. Besides the relative size and spacing of pseudoteeth, irregularities exist also in terms of slant, or even locus of implantation (e.g. in the large *D. emuinus* from Morocco; STable 1).

**Supplementary Discussion, section 11. Interpretable irregularities fit with our model of dynamic inhibition zones.**

Evaluation of irregularities can be attempted through comparison of a variant with a "control" region, especially when the right and left maxillae are available at the same rostro-caudal level. Such a comparison is possible on a fragment from the premaxillary of a *D. toliapicus* from the Paleogene of Morocco (SFig. 4; STable 1). It reveals that at the same rostro-caudal level there is an interval of 20 mm between two PT1s on the left side, whereas the corresponding interval is only 14.3 mm on the right side. The size (base cranio-caudal width) of intervening PT2s is, respectively, 3.3 mm on the left side (80% of PT1) and 2.1 mm on the right side (50% of PT1). There are no significant differences for PT3s. Hence, a longer spacing between two consecutive PT1s corresponds to a larger intervening PT2. This adds support to our model, in which a longer interval implies a wider (and earlier appearing) inhibition-free zone mid-distance between two pseudoteeth, which makes it possible for the pseudotooth developing at this locus to grow for longer and therefore larger.

**Supplementary Discussion, section 12. Evaluating the galloanserine gliding articulation as a factor favouring pseudodentition evolution.**

The "particularities of a galloanserine-like feeding apparatus, such as the gliding jaw joint (e.g. Weber and Hesse 1995)"[40] were suggested as factors favouring the evolution of pseudodentition by Mayr (2011: 458)[29]. The gliding jaw joint is a misleading designation, since the actual feature referred to is a rostro-caudally gliding mandible-quadrate articulation[41,42]. This gliding articulation is present in advanced pseudotoothed birds (*Pelagornis* spp.)[29], at least (unknown because anatomical region is not preserved in fossils of more basal pseudotoothed birds). However, besides Galloanserae and *Pelagornis*, this articulation is also present (true homology unknown) in *Hesperornis* and *Ichthyornis*[29]. Therefore, this feature is probably not strictly linked to increased herbivory (*contra* Weber[41]). Furthermore, there are no means by which this characteristic would preferentially favour or allow pseudotooth evolution. Other characteristics were likely involved in the adaptive

function of pseudodentition, namely the streptognathism and the absence of mandibular symphysis[43] (see Main text). Incidentally, these hypotheses concern adaptive factors possibly favouring the evolutionary emergence and persistence of pseudodentition, and do not pertain to their ontogenetic determinants. As such, these two types of causes, distal and proximal, should not be confused.

## Supplementary Figures

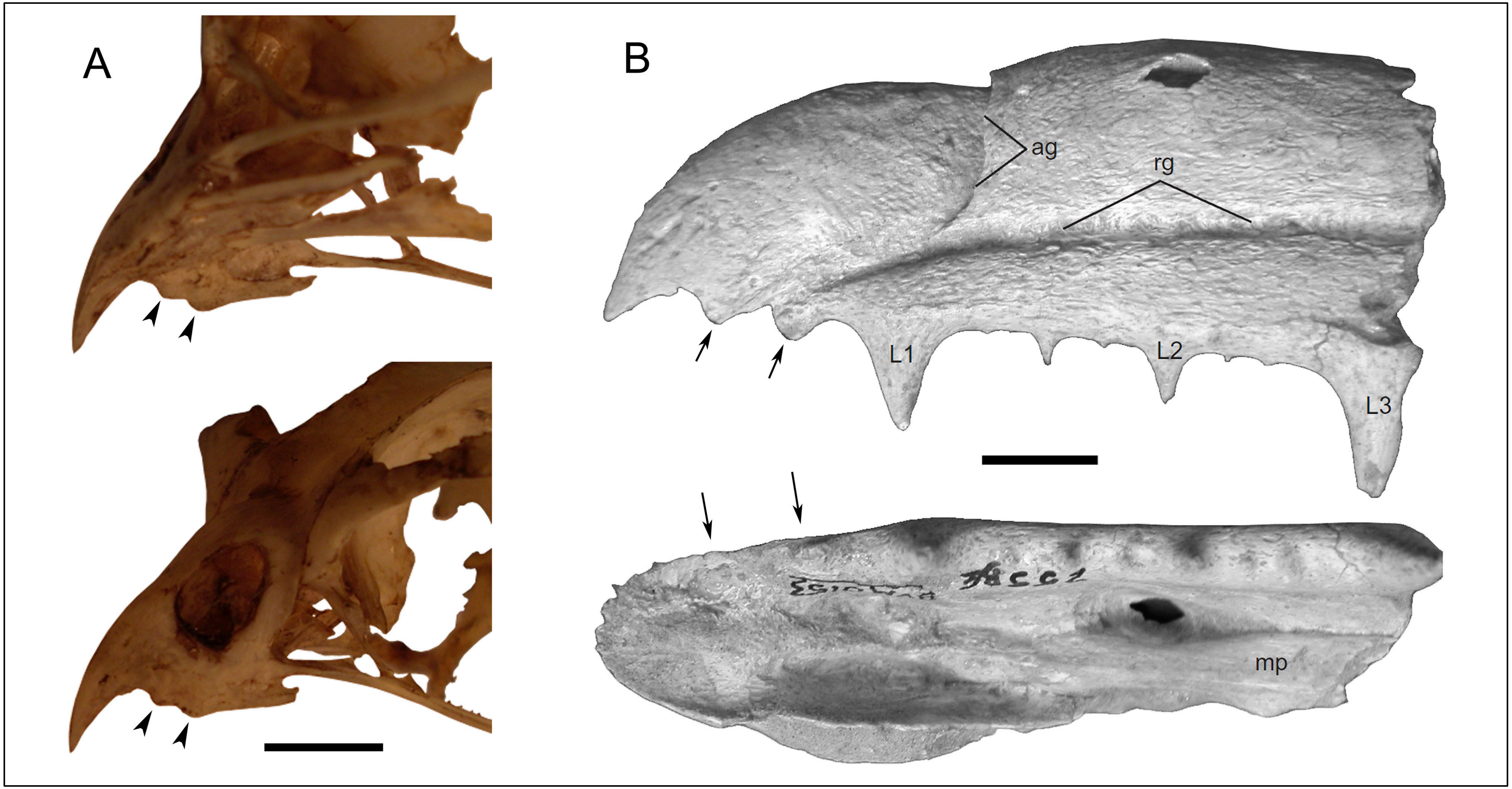


**Supplementary Figure 1.** "Tomial teeth" in an extant bird and a pseudotoothed bird.
**A**, bony rostrum of extant *Harpagus bidentatus* (Aves, Accipitridae), rostral part. Natural light photographs. Top: medio-ventral view of the right pair of "tomial teeth"; bottom: lateral view of the left pair. Arrows indicate the paired "tomial teeth" of *H. bidentatus* that are double, rounded, lateromedially constricted, and located at the rostral tip of the rostrum. These are only bony extensions of the blade-like tomia, and are paralleled by the external shape of the rhamphotheca. **B**, bony rostrum of the extinct *Pelagornis (Osteodontornis) orri* (Aves, Odontopterygiformes), rostral part. Modified from ([22]: fig. 2; *Paleobios*, journal of the Museum of Vertebrate Zoology, University of California Berkeley, USA). Top: left lateral view; bottom: ventral view. These odontopterygiform "tomial teeth" are similar to those of *H. bidentatus*, and other odontopterygiforms (SText 3).

They possibly grew synchronously with the main jaw bone, as opposed to pseudoteeth which grew after the end of the main jaw growth (see SText 3). ag, anterior groove; mp, medial palatal ridge; rg, rostral groove; L1-3, large pseudoteeth 1-3 (all nomenclature after Stidham[22]). Scale bars, 10 mm.

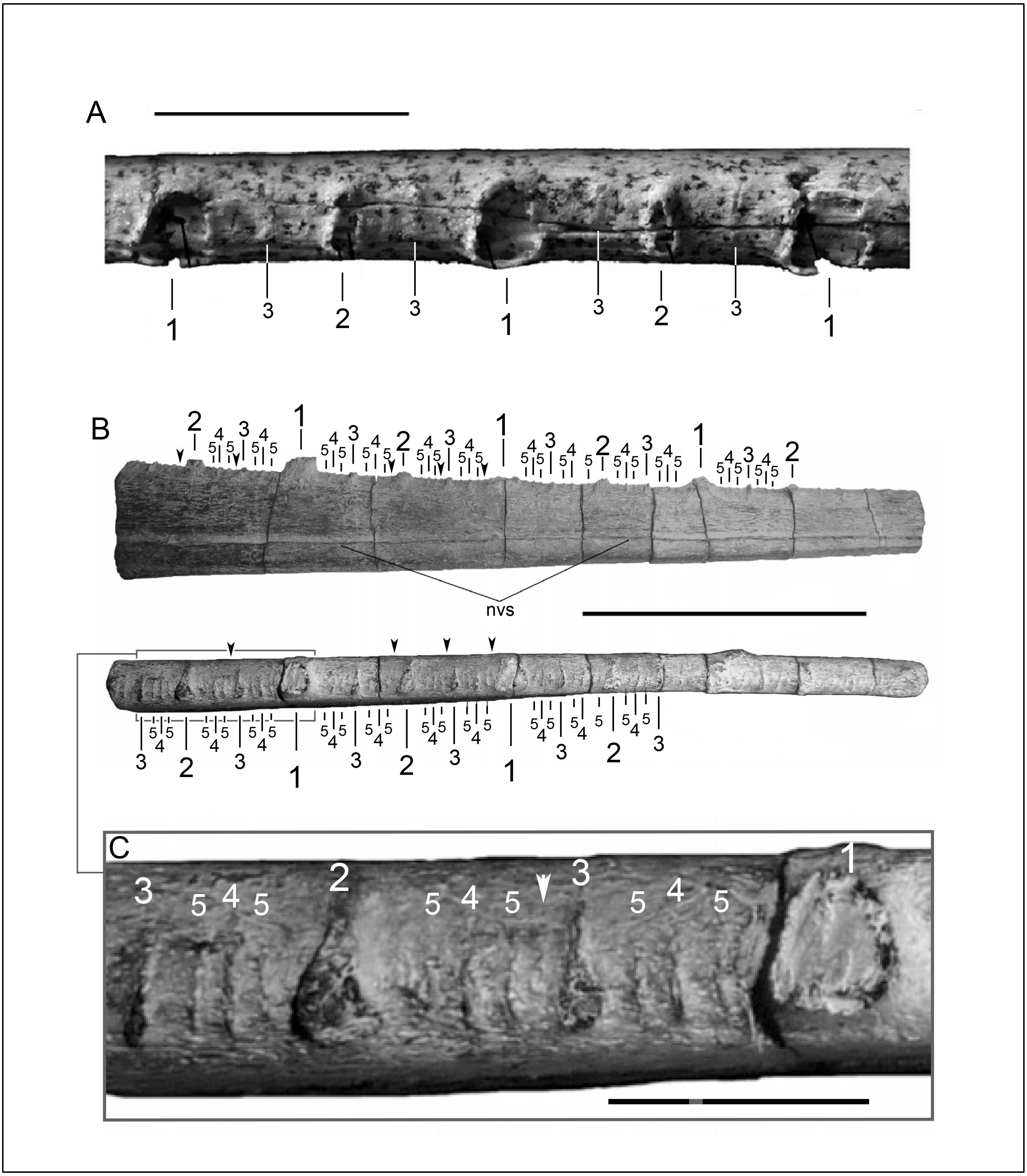


**Supplementary Figure 2.** Juvenile pseudodentitions reveal growth sequence of pseudoteeth.
**A**, Rostral part of right mandibular ramus of juvenile pseudotoothed bird *Lutetodontopteryx tethyensis* from the Middle Eocene of Ukraine, modified from ([30]: fig. 2R; *Journal of Vertebrate Paleontology*, Taylor & Francis Ltd., http://www.informaworld.com). Dorsal view showing broken pseudoteeth 1 and 2 (fully, or almost, grown, owing to their width), and PT3s at bump stage (only starting growth). The latter later developed into PT3s visible between PT1s and PT2s in adults of the same species. **B**, right mandibular ramus in a juvenile of

cf. *Pelagornis* sp., from the Early Miocene of Venezuela, modified from ([23]: fig. 2B,C; *Journal of Vertebrate Paleontology*, Taylor & Francis Ltd., http://www.informaworld.com); lateral (top) and occlusal view (bottom). The rank of pseudoteeth is indicated by numbers 1 to 5. Insert **C**, magnification of the area indicated in (**B**), illustrating the difference between pseudoteeth broken at their base, and bumps (pseudoteeth at an early growth stage), modified from ([23]: fig. 2C). In **A**, **B** and **C**, figure parts from [23] and [30] reprinted, with modification, by permission of the Society of Vertebrate Paleontology, www.vertpaleo.org. This figure is not covered by the CC BY licence. Credits to copyright-holder, Taylor & Francis Ltd. for panels S2A, S2B and S2C. All rights reserved, used with permission. In (**B**) and (**C**), five ranks occur (fourth and fifth at bump stage), a case unique in pseudotoothed birds. In addition, intervening bumps can even be identified irregularly (arrowheads). In (**B**) and (**C**) the pseudodentition is at a comparatively more advanced growth stage than in (**A**), since broken PT3s are fully grown (almost like PT1s and PT2s), and not simple bumps; in (**B**) and (**C**) PT4s and PT5s are at the bump stage, and in (**A**) the PT3s are at bump stage. The juvenile status of both fossils is obvious because of the fibrous aspect of the bone surface. These fossils clearly indicate that lower-ranked (smaller) pseudoteeth start growing later than higher-ranked (larger) ones (SText 7). The two fossils also illustrate the rostro-caudal constriction of pseudoteeth, visible since the earliest growth stages, and increasing with rank. Scale bars, 10 mm (**A**), 50 mm (**B**), 10 mm (insert **C**).

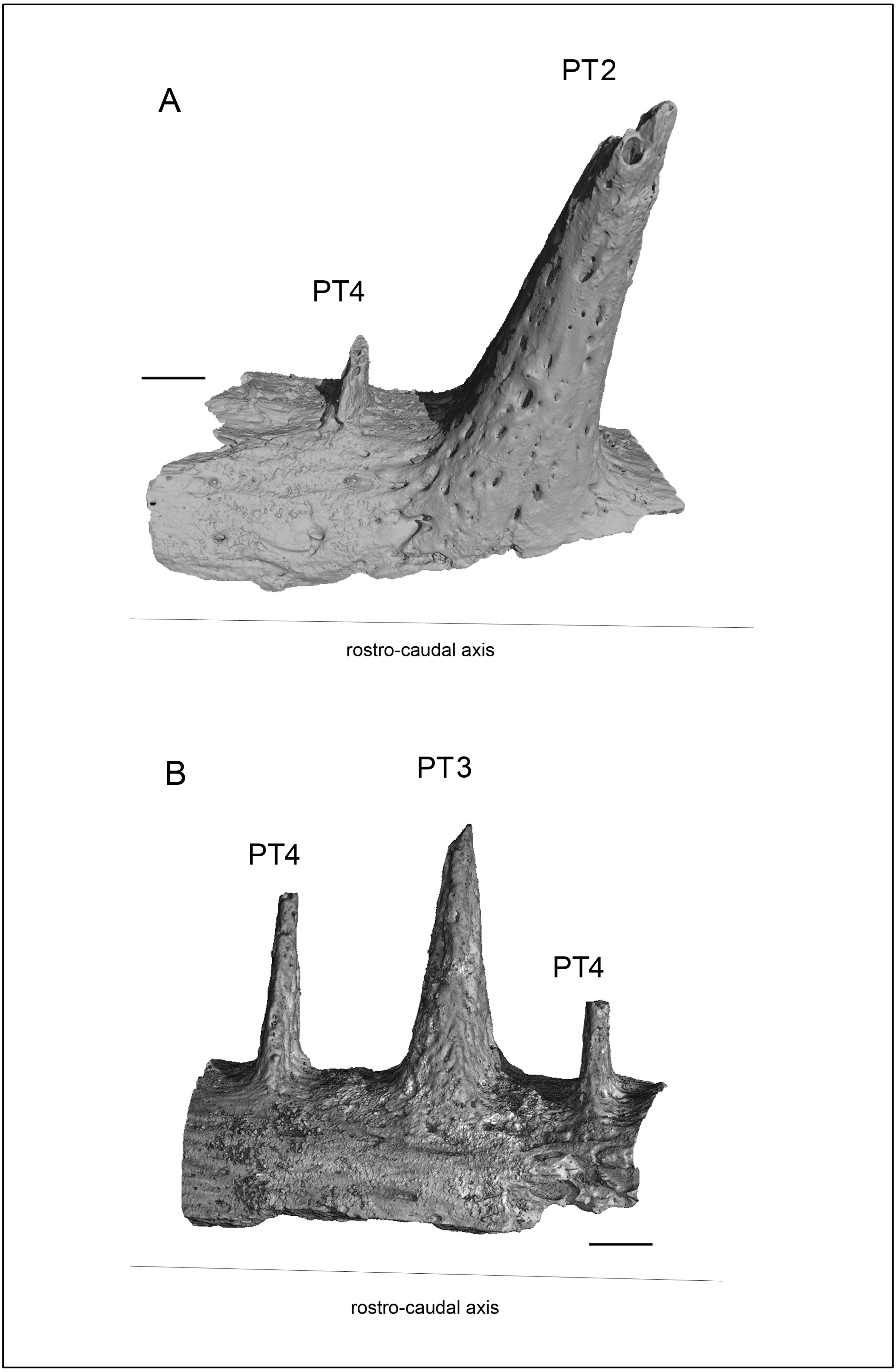


**Supplementary Figure 3**. Pseudoteeth of *Pelagornis mauretanicus* as seen in X-ray microtomographic views. **A**, specimen AaO-PT-B in medial-apical view, and **B**, specimen AaO-PT-A in lateral view[11]. Increasingly lower-rank (smaller) pseudoteeth are increasingly constricted rostro-caudally (the most constricted are the PT4s) (see S.Text 9). The numbers indicate the rank of each pseudotooth. In all the pseudoteeth, the apex is

broken to varying degrees, due to post-mortem breakage; in (**B**) the right PT4 lacks more of the apex than the left PT4. Scale bars, 2 mm.

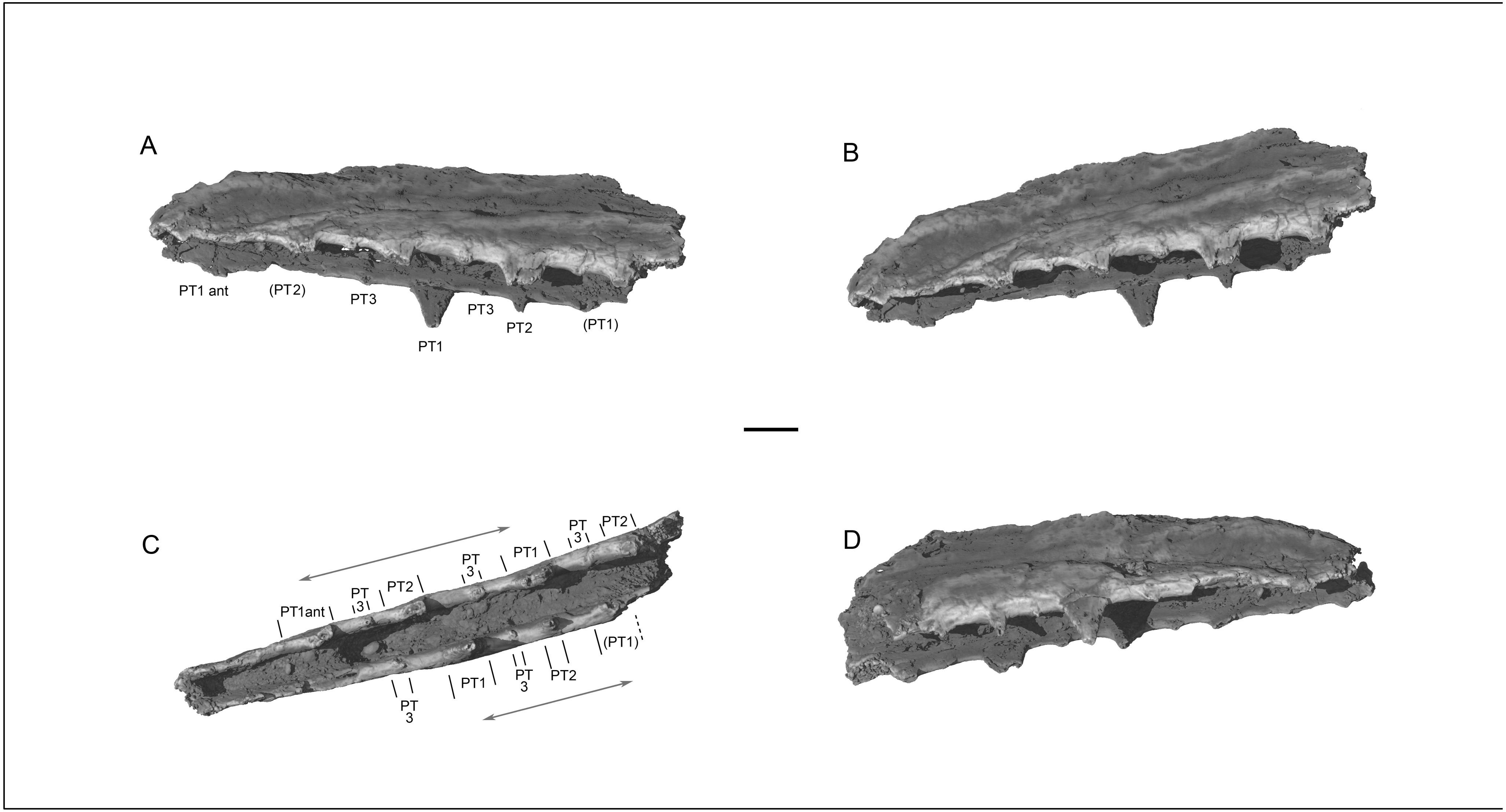


**Supplementary Figure 4**. Rostrum of *Dasornis toliapicus* from the Paleogene of Morocco as seen in X-ray microtomographic views. Distribution and shape of the pseudoteeth of various ranks, on left vs right sides. **A**, **B**, left latero-ventral views; **C**, ventral view; **D**, right latero-ventral view. Increasingly lower-ranked (smaller) pseudoteeth are increasingly constricted rostro-caudally (see SText 9). Spacing between PT1s (grey lines with arrows in **C**) is wider on the left side than on the right, and the intervening PT2 is larger on the left side, which adds support to our dynamic inhibition zone model (see SText 11). Scale bar, 5 mm.

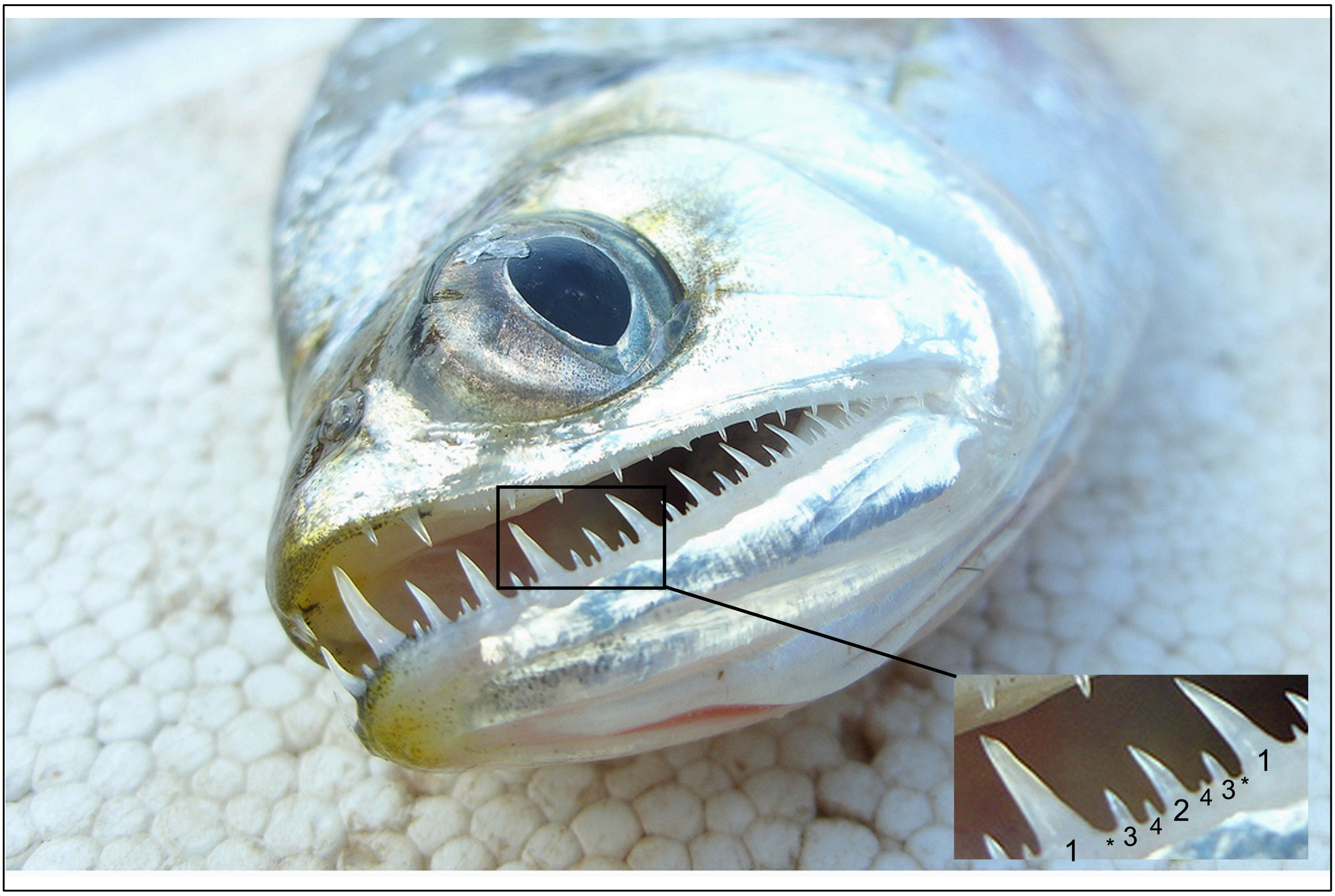


**Supplementary Figure 5**. Dentition of *Cynodon gibbus* (Agassiz, 1829) (Order Characiformes, Family Cynodontidae).
Copyright Adam Carvalho. Modified from: https://www.flickr.com/photos/sickilla/5169310782/
Natural light photograph. Insert shows size classes of true teeth (numbers), arranged exactly as in a pseudodentition. The size distribution of teeth parallels that of pseudoteeth in the odontopterygiform species, in this case with up to four size ranks, though two “rank 4” teeth are lacking in the example (locations indicated by asterisks).

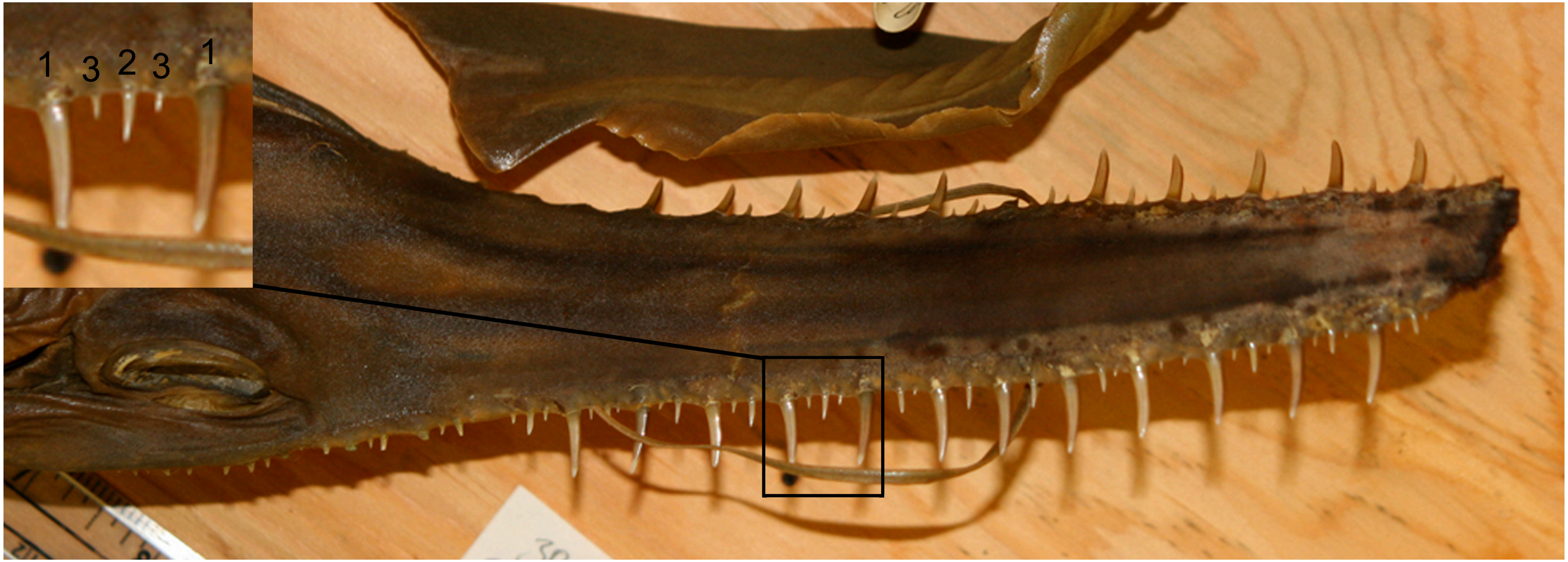


**Supplementary Figure 6**. Dentition of the "saw" (rostrum) of the saw-shark *Pristiophorus cirratus* (Latham, 1794) (Order Pristiophoriformes, Family Pristiophoridae). Copyright Museum of Comparative Zoology, Harvard University, USA. Modified from:
http://mczbase.mcz.harvard.edu/specimen_images/fish/large/38611_Pristiophorus_cirratus_rostrum.jpg
Source URL: https://mczbase.mcz.harvard.edu/guid/MCZ:Ich:38611 ; taxonomic search on https://mczbase.mcz.harvard.edu/
Natural light photograph. Insert shows size classes of true teeth (numbers) that are arranged exactly as in a pseudodentition. In this example, the dentition parallels a three-ranked pseudodentition.

## Supplementary Table

| Species (/specimen) | PT1 cranio-caudal width at base (not too cranial or caudal PT1s) | PT1 height (not too cranial or caudal PT1s) | PT1 cranio-caudal width at base/height ratio (robustness) | interval between PT1s | beak length | (PT1 base cranio-caudal width/beak length) X 100 (%) | length from naso-frontal hinge to caudal end of skull | (PT1 base cranio-caudal width/length from naso-frontal hinge to caudal end of skull) X 100 (%) | PT1 base cranio-caudal width/intervening space between PT1s | PT1 height/intervening space between PT1s | PT2 base cranio-caudal width/PT1 base cranio-caudal width ; PT3 base cranio-caudal width/PT2 base cranio-caudal width ; [and for 4 ranked species:] PT4 base cranio-caudal width/PT3 base cranio-caudal width | PT2 base cranio-caudal width/interval between surrouding PTs ; PT3 base cranio-caudal width/interval between surrouding PTs ; [and for 4 ranked species:] PT4 base cranio-caudal width/interval between surrouding PTs | partly discriminant PT shape attributes | slant of PTs (or absence) | normal distribution of PT ranks | irregularities | geological age | number of specimens (MNI) with jaw parts | references |
|---|---|---|---|---|---|---|---|---|---|---|---|---|---|---|---|---|---|---|---|
| ***Pelagornis mauretanicus*** | 8.0-9.0 | 16.0-20.0 | 0.45-0.5 | 37.0-42.0 (mean 39.8; n=4) | - | - | - | - | 0.21 | 0.45 | 0.6-0.7; 0.5; 0.3-0.4 | 0.224-0.245; 0.106-0.14; 0.09-0.12 | PTs slender, conical, not recurved ; PTs 1 and (2) with caudo-lateral ridge | PT4s vertical, but larger PTs progressively slant forward: mandible PTs ca. 6° to 15° ; rostrum PTs ca. 5° (rostral end PTs) | 1-4-3-4-2-4-3-4-1 | minimal: only in height of some PTs, but not in their presence/position | late Pliocene-early Pleistocene limit | 1 | 11,27 |
| ***Pelagornis stirtoni*** | 5.7 | 14.3 | 0.4 | ca 25.0 | - | - | - | - | 0.23 | 0.57 | ca. 0.4; ?0.5 | - | PTs slender, slightly recurved; PT3s described as spines; no caudo-lateral ridge visible | slant forward: ca 15° (mandible PTs, rostral end) | 1-3-2-3-1 | | Miocene/Pliocene | 1 | 44 |
| ***Pelagornis chilensis*** | 12.5-15.6 | 21.9-25.5e | 0.49-0.65 | 53.9-76.0 (mean 61.5; n=5) | 361.0 | 4.0-4.4 | 144.0 | 10.1-11.0 | 0.23 | 0.39 | 0.5-0.6; 0.4-0.5 | 0.12-0.16 ; 0.12-0.15 | PTs robust, recurved caudally (but variably: rostrum: only the cranialmost ones; mandible: cranial ones and less so the caudal ones); caudo-lateral ridge (rostrum: PT1s, mandible: PT1s and PT2s) | varied slant; mandible PTs: 0° (rostral end) to ca. 15° forward (caudal end); rostrum PTs: ca -18° (ie backwards; rostral end) to 0° (middle) to ca +18° (ie forward; caudal end) | 1-3-2-3-1 | intercalation of a small, supplementary PT (equivalent to a PT4) at least at 5 locations of rostrum between PT1s and PT3s (Mayr and Rubilar 2010: fig. 1B) | middle Miocene-earliest Pliocene | 1 | 19 |
| ***Pelagornis orri*** | 7.0-12.0 | 13.0-19.5e | 0.52-0.62 | 30.0-40.0 (mean 35; n=4) | 300.0 | 2.9-3.3 | 100.0 | 8.8-10.0 | 0.27 | 0.46 | 0.5-0.6; 0.5-0.7; ca. 0.4 | 0.11 (n=2); 0.11-0.17; 0.07-0.13 | PTs robust, only the cranialmost ones being slightly recurved caudally; no visible caudo-lateral ridge | slight slant forward at caudal part of mandible (ca +10°); slight slant backward for PTs at rostral part of rostrum (ca -5° -8° to -18°) ; other parts: 0° | 1-4-3-4-2-4-3-4-1 | few: only in height of some PTs, but less in their presence/position; eg a PT4 larger than a PT3 and almost as large as a PT2 - and variation in intervals too, within a single specimen (rostrum in Olson 1985: fig. 9; similar example in mandible in Stidham 2004: fig. 4); isolated change in slant in a PT3 in Howard and White (1962: figure 3) : ca -20° vs. 0° | middle and late Miocene | 4 or 5 | 22,32,33,45,46 |
| ***Pelagornis longirostris*** | 13.3-15.9 | ca 21.0e-25.0e | 0.57-0.64 | 55.5-63.5 (mean 58.3; n=4) | - | - | ca. 200.0 | 6.7-8.0 | 0.25 | 0.39 | 0.5-0.6; 0.3-0.4 | 0.15; - | PTs robust, not recurved | slant forward: mandible PTs ca 4°-11°; rostrum PTs no slant visible (but poor preservation) | 1-3-2-3-1: it is clear that traces of PT3s bases are visible, especially on mandible, probably eroded due to poor preservation, and despite assertions to the contrary (Howard 1957, Howard and White 1962, among others] | | ? | 1 | 47,48 |
| ***Pelagornis sandersi*** | 11.3; 8.87; 6.86 | 16.1; 16.9; 13.3; 11.3 | 0.61; 0.67; 0.67; 0.7 | 46.0; 56.5 | 405.0 | 2,2; 1,7; 2,79 | 161.0 | 5,5; 4,3; 7.0 | 0.19; 0.15; 0.20 | 0,29; 0,25; 0.28; 0.30 | 0.53-0.55; 0.52-0.65 | 0.105-0.106; 0.11-0.13 | PTs not recurved; caudo-lateral ridge on larger rank PTs | no slant (vertical) except : mandible PTs slant forward at caudal part 15°; rostrum PTs slant backward for some PTs toward caudal part, up to 5° | 1-(4)-3-(4)-2-(4)-3-(4)-1: PT4s present irregularly but wide portions lack them, which is a feature of the species and not an artifact of preservation | important irregularities | late Oligocene | 1 | 24,43 |
| ***Pelagornis* sp. Japan Miocene** | 8.8 | - | - | 42.4 | - | - | - | - | 0.21 | - | 0.63; 0.5; 0.4 | 0.14; 0.13-0.14; 0.09-0.1 | PTs slightly recurved; apparently no caudo-lateral ridge | slant forward of mandible PTs: ca 8° to 15° (rather rostral part of mandible) | 1-4-3-4-2-4-3-4-1 | | late Early Miocene (17.5-17 Ma) | 1 | 49 |
| ***Caspiodontornis kobystanicus*** | 8.4-8.8 | 16.7- - | 0.5 | 51.0 | ca. 227.0 | 3.7-3.9 | ca. 93.0 | 9.0-9.5 | 0.17 | 0.33 | 0.6; 0.5 | 0.11-0.12; 0.09-0.1 | PTs not recurved | when clearly assessable: no slant visible | 1-3-2-3-1 | alleged great irregularity is in fact due to poor preservation | Oligocene | 1 | 50 |
| ***Lutetodontopteryx tethyensis*** | 4.2-4.6 | 9.3-10.0 | 0.45-0.46 | 17.3-17.9 (mean 17.7; n=3) | - | - | - | - | 0.25 | 0.55 | 0.6; 0.4-0.5 | 0.16-0.18; 0.16-0.18 | PTs slender, not recurved caudally; caudo-lateral ridge absent or faint | slant forward (mandible PTs): 17° | 1-3-2-3-1 | | middle Eocene | 2 (1 ad., 1 juv.) | 30,39 |
| ***?Dasornis* sp. / *?Gigantornis* sp. Ukraine** | 8.5-10.0 | 12.5-15.9 | 0.63-0.68 | 53.7 | - | - | - | - | 0.17 | 0.26 | 0.4; 0.6 | - | PTs robust, not recurved; apparently no caudo-lateral ridge | larger PTs of middle mandible: slant forward ca 10° | 1-3-2-3-1 | few irregularities: e.g., a splitting of the PT2 into two smaller, abnormal "PT2s" between the two PT1s (Mayr and Zvonok 2011: text-fig2A). This suggests that an "abnormal" wide space between the PT1s allowed two close, smaller PT2s in place of a normal single one. | middle Eocene | 2 | 30,39 |
| ***Dasornis emuinus* Morocco** (NB: a wide sulcus medially and ventrally on mandible is unlike other pseudotoothed birds except the ?*Dasornis*/?*Gigantornis* of Ukraine (and absence of sternum known for Moroccan fossils allow possibility of *Gigantornis*) | 7.0-9.0 | - | - | 39.5; 47.5 | - | - | - | - | 0.18 | - | 0.5-0.6; 0.5-0.6 | - | Caudo-lateral ridge present | apparently (PTs broken off) none | 1-3-2-3-1 | two mandibular PTs project dorso-laterally rather than dorsally, in a faint apical tomial groove, from where all PTs emerge, but which locally deviates laterally. | late Paleocene-early Eocene | 1 | 51; present study |
| ***Dasornis toliapica* England** | 2.5 | 5.5 | 0.45 | 15.0 | - | - | - | - | 0.17 | 0.37 | 0.7-0.74; 0.6-0.8 | 0,11; 0,12. 0,13; 0,17. | | strong slant forward (caudal part of rostrum and mandible - the only jaw parts known): ca 27° | 1-3-2-3-1 | | early Eocene | 1 | 51,52 |
| ***Dasornis toliapicus* Morocco** | 4.0-4.3 | 6.0e | 0.67 | 14.8 (right side); 20.0 (left side) (mean 17.4; n=2) | - | - | - | - | 0.21 (large interval: left side); 0.28 (small interval: right side): see Text and Supplementary Text | ca. 0.30 (large interval: left side); ca. 0.41 (small interval: right side): see Text and Supplementary Text | large interval (20, left): 0.8; 0.4-0.5 ; small interval (14.8, right): 0.5; 0.5 | 0.19; 0.11-0.2 | PTs robust, not recurved; caudo-lateral ridge seen on one PT only (PT1). | no slant (at least at rostral part of rostrum and mandible, and caudal part of rostrum, ie differs from English *D. toliapica*) | 1-3-2-3-1 | there is a strong asymmetry between left and right side of rostrum: it is not only a shift of PT positions left vs right, but also different spacings of PTs between the two sides (locally larger intervals on left side). | late Paleocene-early Eocene | 2 or 3 | 51; present study |

**Supplementary Table 1**. Measurements and observations on the pseudodentition in species (or specimens) of pseudotoothed birds for which jaw material is available.
These data do not include parts of jaws in which irregularities or abnormalities occur (except when useful and presented as irregularities *per se*). Also, these data do not concern the rostral and caudal ends of jaws, in which pseudotooth size and spacing decrease compared to those of identical rank pseudoteeth located in the middle part of a raw. Measurements are in millimetres, and taken only where the fossil part is not broken.
e, estimated measurement. -, no data available / no measurement possible. Pseudotooth height is measured from the tomium adjacent to pseudotooth base, to the pseudotooth apex, and orthogonal to the tomium. The intervening space (interval) between two pseudoteeth is measured along the tomium between the mid-points of each pseudotooth basal plate.
Pseudotooth width is measured rostro-caudally (which would correspond to "length" in true tooth measurement methodology). Only adult specimens are included.

## Supplementary References


1. Stettenheim, P. R. The integumentary morphology of modern birds – an overview. *Amer. Zool.* **40**, 461–477 (2000).
2. Rand, A. L. On the spurs of birds' wings. *Wilson Bull.* **66**, 127–134 (1954).
3. Beauchamp, A. J. The ageing of Weka (*Gallirallus australis*) using measurements, soft parts, plumage and wing spurs. *Notornis* **45**, 167–176 (1998).
4. Juhn, M. Spur growth and differentiation in the adult thiouracil-treated fowl. *Physiol. Zool.* **25**, 150–162 (1952).
5. Juhn, M. Functional persistence of embryonic determinations in feathers and late developmental stages in spurs. *Ann. New York Acad. Sci.* **55**, 133–141 (1952).
6. Puchkov, V. F. The development of spur germs in chick embryo. *Arkhiv Anatomii, Gistologii i Embriologii* [in Russian] **76**, 32–41 (1979).
7. Davison, G. W. H. Avian spurs. *J. Zool.* 206, 353–366 (1985).
8. Howard, H. Observations on young tarsometatarsi of the fossil turkey *Parapavo californicus* (Miller). *Auk* **62**, 596–603 (1945).
9. Peters, J. Zum Stand der Hühnerhaltung in der Antike. *Beitr. z. Archäozool. u. Prähist. Anthrop.* **1**, 42–58 (1997).
10. Sadler, P. The use of tarsometatarsi in sexing and ageing domestic fowl (*Gallus gallus* L.), and recognising five toed breeds in archaeological material. *Circaea* **8**, 41–48 (1991).
11. Louchart, A. *et al.* Structure and growth pattern of pseudoteeth in *Pelagornis mauretanicus* (Aves, Odontopterygiformes, Pelagornithidae). *PLoS ONE* **8**, e80372 (2013).
12. Olson, S. L. & James, H. F. Descriptions of thirty-two new species of birds from the Hawaiian Islands: part I. Non-passeriformes. *Ornithol. Monogr.* **45**, 1–88 (1991).
13. Fabrezi, M. & Emerson, S. B. Parallelism and convergence in anuran fangs. *J. Zool. Lond.* **260**, 41–51 (2003).
14. Currey, J. D. Mechanical properties and adaptations of some less familiar bony tissues. *J. Mech. Behav. Biomed.* **3**, 357–372 (2010).
15. Britz, R., Conway, K. W. & Rüber, L. Spectacular morphological novelty in a miniature cyprinid fish, *Danionella dracula* n. sp. *Proc. R. Soc. B* **276**, 2179–2186 (2009).
16. Gaffney, E. S. Comparative cranial morphology of recent and fossil turtles. *Bull. Amer. Mus. Nat. Hist.* **164**, 65–376 (1979).
17. Martill, D. M. A functional odontoid in the dentary of the Early Cretaceous pterosaur *Istiodactylus latidens*: Implications for feeding. *Cret. Res.* **47**, 56–65 (2014).
18. Harrison, C. J. O. & Walker, C. A. A review of the bony toothed birds (Odontopterygiformes): with descriptions of some new species. *Tert. Res. Spec. Paper* **2**, 1–62 (1976).
19. Mayr, G. & Rubilar-Rogers, D. Osteology of a new giant bony-toothed bird from the Miocene of Chile, with a revision of the taxonomy of Neogene Pelagornithidae. *J. Vertebr. Paleont.* **30**, 1313–1330 (2010).
20. Seki, Y. *Structure and mechanical behavior of bird beaks*. PhD Dissertation, University of California, San Diego (2009). Permalink: http://escholarship.org/uc/item/7064f0k0
21. Louchart, A. & Viriot, L. From snout to beak: the loss of teeth in birds. *Trends Ecol. Evol.* **26**, 663–673 (2011).
22. Stidham, T. A. New skull material of *Osteodontornis orri* (Aves: Pelagornithidae) from the Miocene of California. *PaleoBios* **24**, 7–12 (2004).
23. Solórzano, A. & Rincón, A. D. The earliest record (early Miocene) of a bony-toothed bird from South America and a reexamination of Venezuelan pelagornithids, *J. Vertebr. Paleont.* **35**: e995188 (2015).

24. Ksepka, D. T. Flight performance of the largest volant bird. *Proc. Natl Acad. Sci. USA* **111**, 10624–10629 (2014).
25. Reid, K., Prince, P. A. & Croxall, J. P. Fly or die: the role of fat stores in the growth and development of Grey-headed Albatross *Diomedea chrysostoma* chicks. *Ibis* **142**, 188–198 (2000).
26. Granadeiro, J. P. The breeding biology of Cory's Shearwater *Calonectris diomedea borealis* on Berlenga Island, Portugal. *Seabird* **13**, 30–39 (1991).
27. Mourer-Chauviré, C. & Geraads, D. The Struthionidae and Pelagornithidae (Aves: Struthioniformes, Odontopterygiformes) from the late Pliocene of Ahl Al Oughlam, Morocco. *Oryctos* **7**, 169–194 (2008).
28. Harris, M. P., Hasso, S. M., Ferguson, M. W. J. & Fallon, J. F. The development of archosaurian first-generation teeth in a chicken mutant. *Curr. Biol.* **16**, 371–377 (2006).
29. Mayr, G. Cenozoic mystery birds – on the phylogenetic affinities of bony-toothed birds (Pelagornithidae). *Zool. Scr.* **40**, 448–467 (2011).
30. Mayr, G. & Zvonok, E. A new genus and species of Pelagornithidae with well-preserved pseudodentition and further avian remains from the middle Eocene of the Ukraine. *J. Vertebr. Paleont.* **32**, 914–925 (2012).
31. Dumont, M. *et al.* Synchrotron imaging of dentition provides insights into the biology of *Hesperornis* and *Ichthyornis*, the "last" toothed birds. *BMC Evol. Biol.* **16**, 178 (2016).
32. Howard, H. A gigantic ''toothed'' marine bird from the Miocene of California. *Bull. Dept. Geol. Santa Barbara Mus. Nat. Hist.* **1**, 1–23 (1957).
33. Howard, H. & White, J. A. A second record of *Osteodontornis*, Miocene "toothed" bird. *Los Angeles Cnty Mus. Contrib. Sci.* **52**, 1–12 (1962).
34. Westergaard, B. & Ferguson, M. W. J. Development of the dentition in *Alligator mississippiensis*: upper jaw dental and craniofacial development in embryos, hatchlings, and young juveniles, with a comparison to lower jaw development. *Am. J. Anat.* **187**, 393–421 (1990).
35. Sire, J. Y., Davit-Béal, T., Delgado, S., van der Heyden, C. & Huysseune, A. First-generation teeth in non-mammalian lineages: evidence for a conserved ancestral character ? *Microscop. Res. Techn.* **59**, 408–434 (2002).
36. Sorenson, M. D. *et al.* Relationships of the extinct moa-nalos, flightless Hawaiian waterfowl, based on ancient DNA. *Proc. R. Soc. Lond. B* **266**, 2187–2193 (1999).
37. Bourdon, E. Osteological evidence for sister group relationship between pseudo-toothed birds (Aves: Odontopterygiformes) and waterfowls (Anseriformes). *Naturwissenschaften* **92**, 586–591 (2005).
38. Iwaniuk, A. N., Nelson, J. E., James, H. F. & Olson, S. L. A comparative test of the correlated evolution of flightlessness and relative brain size in birds. *J. Zool. Lond.* **263**, 317–327 (2004).
39. Mayr, G. & Zvonok, E. Middle Eocene Pelagornithidae and Gaviiformes (Aves) from the Ukrainian Paratethys. *Palaeontology* **54**, 1347–1359 (2011).
40. Weber, E. & Hesse, A. The systematic position of *Aptornis*, a flightless bird from New Zealand. *Cour. Forsch.-Inst. Senckenberg* **181**, 293–301 (1995).
41. Weber, V. E. Zur Evolution basicranialer Gelenke bei Vögeln, insbesondere bei Hühner- und Entenvögeln (Galloanseres [sic]). *Z. zool. Syst. Evolut-forsch.* **31**, 300–317 (1993).
42. Ericson, P. G. P. The skeletal evidence for a sister-group relationship of anseriform and galliform birds – a critical evaluation. *J. Avian Biol.* **27**, 195–202 (1996).
43. Zusi, R. L. & Warheit, K. I. On the evolution of intraramal mandibular joints in pseudodontorns (Aves: Odontopterygia). *Nat. Hist. Mus. Los Angeles Cnty Sci. Ser.* **36**, 351–360 (1992).

44. Howard, H. & Warter, S. L. A new species of bony-toothed bird (Family Pseudodontornithidae) from the Tertiary of New Zealand. *Rec. Canterbury Mus.* **8**, 345–357 (1969).
45. Howard, H. Additional avian records from the Miocene of Kern County, California with the description of a new species of fulmar (Aves: Procellariidae). *Bull. South. Cal. Acad. Sci.* **83**, 84–89 (1984).
46. Olson, S. L. in *Avian Biology* (eds D. S. Farner, J. R. King & K. C. Parkes) 79–252 (Academic Press, 1985).
47. Spulski, B. *Odontopteryx longirostris* n. sp. *Zeitschrift der deutschen geologischen Gesellschaft* **62**, 507–521 (1910).
48. Lambrecht, K. Studien über fossile Riesenvögel. *Geologica Hungarica, Series Palaeontologica* **7**, 1–37 (1930).
49. Matsuoka, H., Sakakura, F. & Ohe, F. A Miocene pseudodontorn (Pelecaniformes: Pelagornithidae) from the Ichishi Group of Misato, Mie Prefecture, Central Japan. *Paleont. Res.* **2**, 246–252 (1998).
50. Aslanova, S. M. & Burchak-Abramovich, N. I. [An Oligocene pseudodontornithid bird from Perekiškjul' village (Apšeronskij Peninsula)—the first and only record from the USSR and the Asian continent]. *Izvestija Akademii Nauk Gruzinskoj SSR, Seriya Biologicheskayaa* **8**, 406–412 (1982). [Russian]
51. Bourdon, E., Amaghzaz, M. & Bouya, B. Pseudotoothed birds (Aves, Odontopterygiformes) from the early Tertiary of Morocco. *Am. Mus. Novit.* **3704**, 1–71 (2010).
52. Owen, R. Description of the skull of a dentigerous bird (*Odontopteryx toliapicus* [sic], Ow.) from the London Clay of Sheppey. *Quart. J. Geol. Soc. London* **29**, 511–521 (1873).
